# A generalizable 29-mRNA neural-network classifier for acute bacterial and viral infections

**DOI:** 10.1038/s41467-020-14975-w

**Published:** 2020-03-04

**Authors:** Michael B. Mayhew, Ljubomir Buturovic, Roland Luethy, Uros Midic, Andrew R. Moore, Jonasel A. Roque, Brian D. Shaller, Tola Asuni, David Rawling, Melissa Remmel, Kirindi Choi, James Wacker, Purvesh Khatri, Angela J. Rogers, Timothy E. Sweeney

**Affiliations:** 1 Inflammatix, Inc., 863 Mitten Rd, Suite 104, Burlingame, CA 94010 USA; 2 Department of Medicine, Stanford University, Palo Alto, CA 94305 USA; 3 Division of Pulmonary, Allergy, and Critical Care Medicine, Department of Medicine, Stanford University, Palo Alto, CA 94305 USA; 4 Institute for Immunity, Transplantation and Infections, Stanford University, Palo Alto, CA 94305 USA; 5 Center for Biomedical Informatics Research, Department of Medicine, Stanford University, Palo Alto, CA 94305 USA

**Keywords:** Computational biology and bioinformatics, Diagnostic markers, Bacterial infection, Viral infection

## Abstract

Improved identification of bacterial and viral infections would reduce morbidity from sepsis, reduce antibiotic overuse, and lower healthcare costs. Here, we develop a generalizable host-gene-expression-based classifier for acute bacterial and viral infections. We use training data (*N* = 1069) from 18 retrospective transcriptomic studies. Using only 29 preselected host mRNAs, we train a neural-network classifier with a bacterial-vs-other area under the receiver-operating characteristic curve (AUROC) 0.92 (95% CI 0.90–0.93) and a viral-vs-other AUROC 0.92 (95% CI 0.90–0.93). We then apply this classifier, inflammatix-bacterial-viral-noninfected-version 1 (IMX-BVN-1), without retraining, to an independent cohort (*N* = 163). In this cohort, IMX-BVN-1 AUROCs are: bacterial-vs.-other 0.86 (95% CI 0.77–0.93), and viral-vs.-other 0.85 (95% CI 0.76–0.93). In patients enrolled within 36 h of hospital admission (*N* = 70), IMX-BVN-1 AUROCs are: bacterial-vs.-other 0.92 (95% CI 0.83–0.99), and viral-vs.-other 0.91 (95% CI 0.82–0.98). With further study, IMX-BVN-1 could provide a tool for assessing patients with suspected infection and sepsis at hospital admission.

## Abstract (web summary)

Diagnosing acute infections based on transcriptional host response shows promise, but generalizability is wanting. Here, the authors use a co-normalization framework to train a classifier to diagnose acute infections and apply it to independent data on a targeted diagnostic platform.

## Introduction

Severe acute infections and sepsis are globally associated with substantial mortality (nearly half of all inpatient deaths) and dollars spent ($24 billion annually in the US)^1–3^. While early antibiotics for patients with sepsis saves lives, inappropriate use of antibiotics can cause morbidity, increased costs, and antimicrobial resistance^4,5^. Thus, current sepsis guidelines and mandates emphasize antibiotic treatment within 1 h, but this has led to significant concern about overtreatment and a need for improved diagnostics^6,7^. Microbiological cultures are the gold standard for bacterial identification, but they are slow, susceptible to contamination, and are negative in roughly 40–60% of patients hospitalized for acute infections and sepsis^8,9^.

An alternative to testing for pathogens is to examine the host immune response to infection, and thereby infer the presence and type of infection^10^. Recent approaches to multi-mRNA diagnostic panels have used simple statistical models to integrate multiple targets into a single diagnostic score^11–16^. Recent advances in machine learning and artificial intelligence offer the promise both of improved generalizability and of solving non-binary problems, such as distinguishing between bacterial, viral, and non-infectious inflammation.

Historically, applying machine learning to diagnose acute infections using transcriptomic data has been confounded by technical and clinical heterogeneity in attempts to translate to real-world patient populations. For example, regression and decision tree classifiers trained using data collected on one type of microarray and tested in another type perform poorly, arguably at least in part due to inadequate cross-platform normalization^13,17^. Even models tested in data from the same technical platform can be prone to overfitting due to the lack of adequate representation of clinical heterogeneity in the training data^18^. We have repeatedly demonstrated that leveraging biological and technical heterogeneity across a large number of studies taken from diverse clinical backgrounds and profiled using different platforms increases generalizability required for clinical translation^11–13,17,19–22^. Ideally, a classifier could be trained across multiple representative clinical studies, in concert with appropriate methods for data co-normalization, such as COmbat CO-Normalization Using conTrols (COCONUT)^12,23^. However, although such a classifier may be generalizable, it still has to be adapted to the gene expression measurements of a purpose-built diagnostic instrument to be useful in a clinical setting.

We have previously described three non-overlapping host response-based mRNA scores that could (1) diagnose the presence of an acute infection (11 mRNAs)^11^, (2) distinguish it as bacterial or viral (7 mRNAs)^12^, and (3) determine the risk of 30-day mortality from sepsis (12 mRNAs)^13^. In this work, we demonstrate that by starting with these preselected variables and applying a novel co-normalization framework to match transcriptomic data onto a targeted diagnostic platform, we can train a generalizable machine-learning classifier for diagnosing acute infections (IMX-BVN-1; IMX—Inflammatix, BVN—bacterial-viral-noninfected, version 1). Our results could have profound implications not just in improved clinical care in acute infections and sepsis, but also more broadly in machine-learning-based multi-cohort diagnostic development.

## Results

### Preparation of IMX training data

An overall study schema is presented in Supplementary Fig. 1. Our search identified 18 studies (*N* = 1069 patient samples) which met our inclusion criteria, comprising adult patients from a wide range of geographical regions, clinical care settings and disease contexts (Table 1)^15,24–38^. The 29 genes of interest from these studies were co-normalized and then aligned to NanoString mRNA expression values for 40 commercial healthy controls (Supplementary Fig. 2). The resulting NanoString-aligned dataset, which does not include any of the healthy controls, was designated “IMX”.

**Table 1:** Characteristics of training studies.

Study identifier	First author	Study description	Timing of diagnosis	*N*	Age	Male	Severity	Platform	Country	Bacterial	Viral	Noninfected
E-MEXP-3589	Almansa	Patients hospitalized with COPD exacerbation	Hospital/ICU admission	23	70.1^a^	18 (78)	unk.	Agilent	Spain	4 (17)	5 (22)	14 (61)
E-MTAB-1548	Almansa	Surgical patients with sepsis (EXPRESS)	Average post-operation day 4	140	69.7 (± 13.1)	96 (69)	APACHE II 17.0 (±5.4)	Agilent	Spain	82 (59)	0	58 (41)
E-MTAB-5273 / 5274	Burnham	Sepsis due to faecal peritonitis or pneumonia	Within one day of ICU admission	228	65.4 (±15.5)	128 (56)	APACHE II 17.1^a^, SOFA 6.2^a^	Illumina	UK	228 (100)	0	0
GSE13015 (GPL6106)	Pankla	Sepsis, many cases from burkholderia	Within 48 h of diagnosis; both community-acquired and hospital-acquired	45	54.1 (±11.5)	26 (58)	Survivor: 32; Non-survivor: 13	Illumina	Thailand	45 (100)	0	0
GSE13015 (GPL6947)				15	53.5 (±12.3)	6 (40)	Survivor: 8; Non-survivor: 7			15 (100)	0	0
GSE20346	Parnell	Influenza and bacterial pneumonia in ICU	“At peak symptoms”	10	51 (range 21-75)	4 (40)	Apache II 18.8 (range 10-33)	Illumina	Australia	6 (60)	4 (40)	0
GSE21802	Bermejo-Martin	Pandemic H1N1 in ICU	Within 48 h of ICU admission	12	unk.	6 (50)	SOFA 3.7	Illumina	Canada	0	12 (100)	0
GSE27131	Berdal	Severe H1N1	Admission to ICU	7	41.1 (±12.7)	6 (86)	SAPS II 29.3 (±11.2)	Affymetrix	Norway	0	7 (100)	0
GSE28750	Sutherland	Sepsis or post-surgical SIRS	Admission to ICU	21	unk.	11 (52)	unk.	Affymetrix	Australia	10 (48)	0	11 (52)
GSE40012	Parnell	Bacterial or influenza A pneumonia or SIRS	Admission to ICU	34	55.4^a^	19 (56)	APACHE II 16.7^a^	Illumina	Australia	16 (47)	6 (18)	12 (35)
GSE42834	Bloom	Bacterial pneumonia or sarcoidosis	Unk.	82	unk.	42 (51)	unk.	Illumina	UK, France	14 (17)	0	68 (83)
GSE57065	Cazalis	Septic shock	Admission to ICU	28^b^	Median 62 (IQR 54-76)	19 (68)	SAPS II median 45 (IQR 34-56); SOFA median 10 (IQR 9-13)	Affymetrix	France	82^b^ (100)	0	0
GSE60244	Suarez	Lower respiratory tract infections	Within 24 h of admission	93	62.2 (±18.1)	37 (40)	unk.	Illumina	USA	22 (24)	71 (76)	0
GSE65682	Scicluna	Suspected but negative for CAP	Within 24 h of ICU admission	33	55.2 (±17.2)	22 (67)	APACHE IV median 74 (IQR 49-112)	Affymetrix	Netherlands	0	0	33 (100)
GSE68310	Zhai	Outpatients with acute respiratory viral infections	Within 48 h of onset	104	unk.	50 (48)	unk.	Illumina	USA	0	104 (100)	0
GSE69528	Khaenam	Sepsis, many cases from burkholderia	Unk.	83	unk.	39 (47)	unk.	Illumina	Thailand	83 (100)	0	0
GSE82050	Tang	Moderate and severe influenza infection	Within 24 h of admission	24	60.8 (±19.6)	14 (58)	ICU: 5 (21); Mech.vent.: 4 (16%	Agilent	Germany	0	24 (100)	0
GSE111368	Dunning	Influenza H1N1 and B	At recruitment	33	38.3 (±12.8)	15 (45)	No SupplementaryO2: 16 (48); O2 by mask: 11 (33); Mech. Vent.: 6 (18)	Illumina	UK	0	33 (100)	0

Numbers in parentheses for Male sex and infection status indicate percentages.

COPD, chronic obstructive pulmonary disorder; TB, tuberculosis; Unk., unknown; ICU, intensive care unit.

^a^ Study description is taken from the study’s corresponding publication and includes some patients that were excluded from IMX.

^b^ Study includes 28 patients assayed at admission and at 24 h and/or 48 h post admission; all 82 time-points were included in IMX.

We visualized IMX using t-distributed stochastic neighbor embedding and principal component analysis^39^. We observed broad class separability (bacterial, viral, or noninfected) but also residual study heterogeneity even after correction for technical batch effects with COCONUT **(**Supplementary Fig. 3). Some of this residual heterogeneity is expected, owing to the clinical heterogeneity inherent across sepsis cohorts^40–42^, but highlighted the need for a robust training procedure^43^.

### Leave-one-study-out (LOSO) cross validation (CV) shows less bias than k-fold CV

**Fig. 1:**
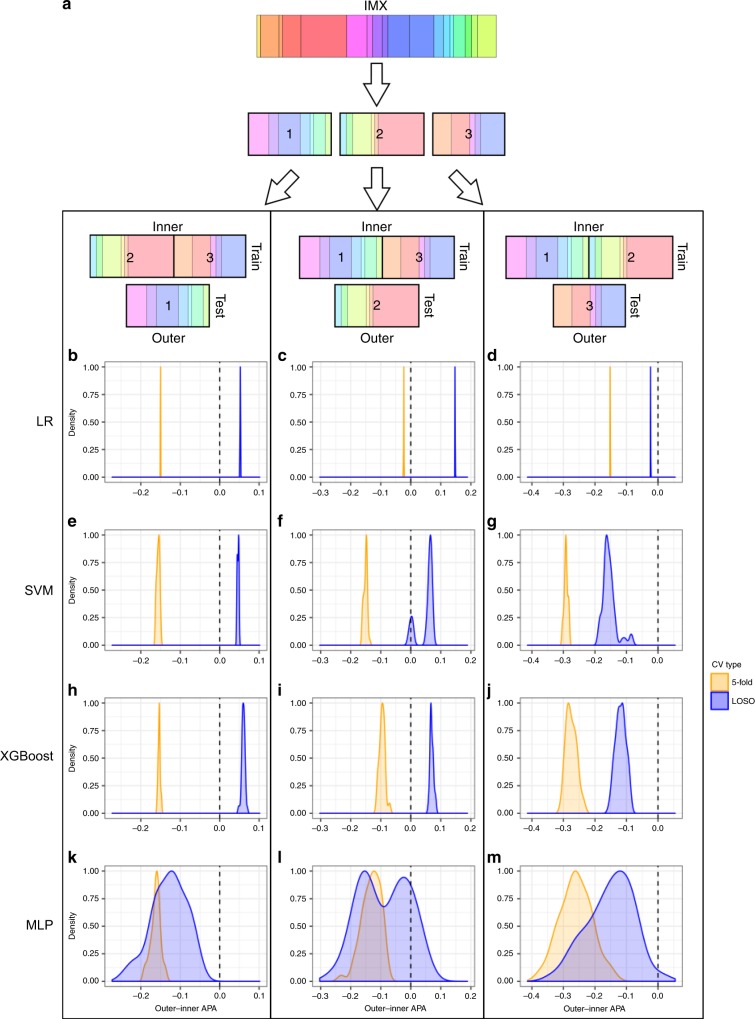
HiCV schematic and results. **a** schematic of hierarchical cross-validation (HiCV). The 18 studies (colored bands) of the IMX dataset are initially partitioned (first arrow) into three roughly equal groups of studies or folds. To simulate model selection and external validation, two of the three folds (inner) are grouped (second set of arrows) and used for cross-validation and training with the remaining fold (outer) used as a test set. This procedure is performed three times, once with each of the partitions of the IMX data treated as a test set. **b**–**m** HiCV analysis of bias/overfitting using 6-GM scores. **b**–**d** LR, logistic regression; (**e**–**g**) SVM, support vector machine; (**h**–**j**) XGBoost, extreme gradient-boosted trees; (**k**–**m**) MLP, multi-layer perceptrons. Each row contains HiCV results for outer folds 1 (**b**, **e**, **h**, **k**), 2 (**c**, **f**, **i**, **l**) or 3 (**d**, **g**, **j**, **m**). The *x*-axis is the difference between outer fold APA and inner fold CV APA. The blue density plots correspond to this difference for the top 50 models ranked by LOSO CV on the inner fold. Orange density plots show this difference for the top 50 models ranked by 5-fold CV on the inner fold. The vertical dashed line indicates equality between inner fold and outer fold APA (low bias), and density plots closer to this line highlight CV methods that potentially result in classifiers with lower generalization bias. Negative values indicate that inner fold APA was higher than outer fold APA, suggesting overfitting during training.

For hierarchical CV (HiCV), we partitioned the IMX dataset into three folds with similar compositions of bacterial, viral and noninfected samples, where any given study appeared in only one fold (Fig. 1a, Supplementary Table 1). We determined which CV type (k-fold vs. LOSO) and feature type (29-mRNA vs. 6-GM) to use in our classifier development with the HiCV schema. Higher average pairwise AUROC (APA) in inner folds compared to the corresponding outer fold (high bias) suggest that the given CV method (or input feature type) results in models prone to overfitting the inner fold data. We found that k-fold CV APA on the inner folds fell substantially in the outer folds, while LOSO CV showed a much smaller difference between inner and outer fold APA for either 6-GM scores or 29-mRNA inputs (Fig. 1 and Supplementary Fig 4**)**. In most cases, LOSO CV also produced models with a higher absolute outer-fold APA (Supplementary Figs. 5–6). These results demonstrate that LOSO CV may produce classifiers with better generalizability to unseen data. Further, LOSO CV outer-fold APA was higher using the 6-GM scores as features rather than the 29-mRNA expression values (Supplementary Table 2).

### Final classifier development

**Fig. 2:**
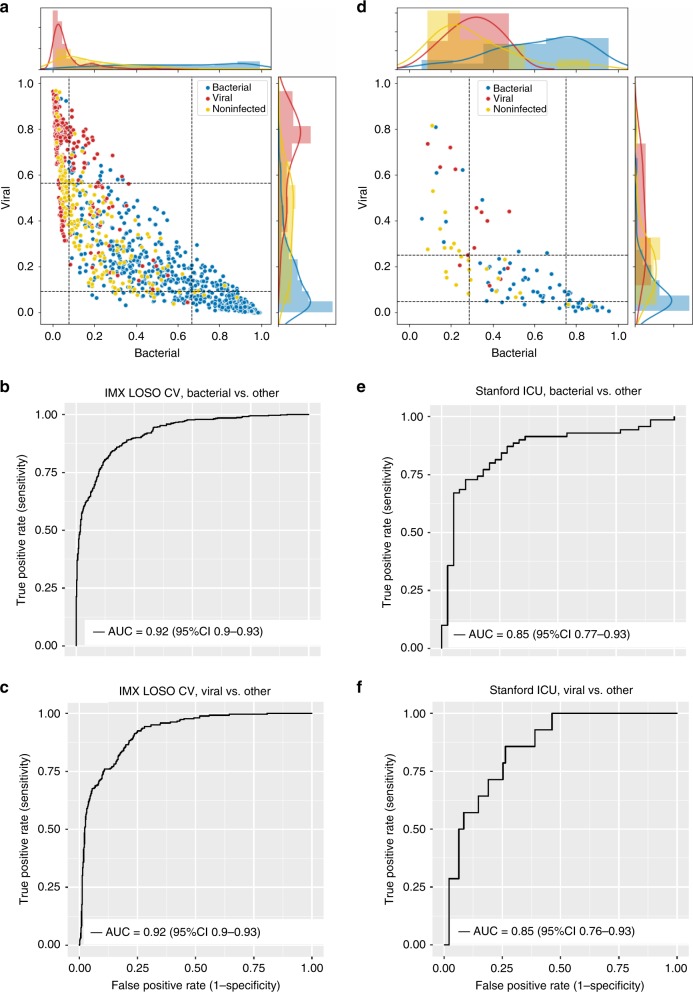
Distribution of IMX-BVN-1 predicted bacterial and viral probabilities in IMX LOSO and Stanford ICU validation. **a**,**d** Each dot in the scatter plot corresponds to a sample (*x*-axis: bacterial predicted probability, y-axis: viral predicted probability). The histogram/density plot above the scatter plot shows bacterial probabilities while the plot to the right of the scatter plot shows viral probabilities. The dotted lines indicate cutoffs for the lower and upper quartiles. **b**, **c**, **e**, **f** Receiver-operating characteristic (ROC) curves for IMX-BVN-1 in IMX (**b**,**c**) and Stanford ICU (**e**,**f**) data for bacterial-vs-other (**b**,**e**) and viral-vs-other (**c**,**f**) comparisons. AUC, area under the ROC curve.

Based on our HiCV analysis, we used LOSO CV and the 6-GM scores on the whole IMX dataset to create a final classifier. We performed hyperparameter searches for logistic regression (LR), support vector machine (SVM), extreme gradient-boosted trees (XGBoost), and multi-layer perceptrons (MLP) models. The best LOSO CV APA results for the full IMX dataset and the four model types were: 0.76, 0.85, 0.77, and 0.87 for LR, SVM, XGBoost, and MLP, respectively. We selected MLP based on its highest ranking in LOSO CV APA. The best performing MLP hyperparameter combination was a two-hidden-layer, four-nodes-per-layer architecture with linear activations at each hidden layer. The model was trained in 250 iterations, with a learning rate of 1e−5, batch normalization^44^, and lasso regularization with a penalty coefficient of 0.1. The MLP had a bacterial-vs.-other area under the receiver-operating characteristic curve (AUROC) of 0.92 (95% CI 0.90–0.93), a viral-vs.-other AUROC of 0.92 (95% CI 0.90–0.93), and a noninfected-vs.-other AUROC of 0.78 (95% CI 0.75–0.81) in LOSO CV (Fig. 2a–c).

To generate a final neural-network model for use in prospective clinical studies, we trained an MLP on all IMX data using the best-performing hyperparameter configuration. Weights and parameter values of this model were fixed after training on the IMX dataset with no subsequent modification or NanoString-specific adjustment. We named this final “fixed-weight” classifier “IMX-BVN-1” (InflaMmatiX Bacterial-Viral-Noninfected, version 1). IMX-BVN-1 generalizes to independent Stanford intensive care unit (ICU) cohort.

We next tested the fixed IMX-BVN-1 classifier in an independent clinical cohort (the Stanford ICU Biobank) run on NanoString nCounter (Table 2; Supplementary Fig. 7**;** Supplemental Data). Across all patients with unanimous infection adjudications (those with 3/3 votes for bacterial, viral, or non-infectious status, *N* = 109), IMX-BVN-1 had a bacterial-vs.-other AUROC of 0.86 (95% CI 0.77–0.93), a viral-vs.-other AUROC of 0.85 (95% CI 0.76–0.93), and a noninfected-vs.-other AUROC of 0.82 (95% CI 0.70–0.91) (Fig. 2d–f). IMX-BVN-1 is intended for use near the time of suspicion of infection; importantly, in patients enrolled within 36 h of hospital admission (*N* = 70), IMX-BVN-1 had a bacterial-vs.-other AUROC of 0.92 (95% CI 0.83–0.99), a viral-vs.-other AUROC of 0.91 (95% CI 0.82–0.98), and a noninfected-vs.-other AUROC of 0.86 (95% CI 0.72–0.96). This almost identical AUROC to the IMX LOSO CV results suggest little-to-no bias/overfitting in IMX-BVN-1. We further tested IMX-BVN-1 according to the presence of immuno-compromise, and showed no significant difference in diagnostic power in this subgroup (Supplementary Table 3).

**Table 2:** Demographic and clinical characteristics of the Stanford ICU patients.

	Noninfected	Bacterial infection (micro positive & consensus)	Viral infection	Noninfected (forced adjudication)	Bacterial infection (forced adjudication)	Fungal infection	Mixed Infection	*P* value
*n*	25	70	14	26	12	2	14	
Age (years)	61.6 (19.0)	66.3 (17.0)	57.4 (15.1)	63.0 (16.3)	71.7 (7.8)	33.5 (0.7)	64.9 (11.7)	0.031
Female sex (%)	11 (44.0)	32 (45.7)	5 (35.7)	10 (38.5)	6 (50.0)	1 (50.0)	7 (50.0)	0.977
Self-identified race/ethnicity (%)								0.25
White	15 (60.0)	46 (65.7)	9 (64.3)	15 (57.7)	5 (41.7)	0 (0.0)	6 (42.9)	
Black/African-American	4 (16.0)	1 (1.4)	0 (0.0)	2 (7.7)	0 (0.0)	0 (0.0)	1 (7.1)	
Asian	1 (4.0)	4 (5.7)	1 (7.1)	0 (0.0)	1 (8.3)	0 (0.0)	2 (14.3)	
Hispanic	0 (0.0)	2 (2.9)	0 (0.0)	1 (3.8)	0 (0.0)	0 (0.0)	1 (7.1)	
Native Hawaiian or Pacific Islander	1 (4.0)	3 (4.3)	0 (0.0)	2 (7.7)	2 (16.7)	0 (0.0)	2 (14.3)	
Other	1 (4.0)	0 (0.0)	1 (7.1)	0 (0.0)	0 (0.0)	0 (0.0)	1 (7.1)	
Unknown	3 (12.0)	14 (20.0)	3 (21.4)	6 (23.1)	4 (33.3)	2 (100.0)	1 (7.1)	
ED admission (%)	20 (80.0)	53 (75.7)	11 (78.6)	17 (65.4)	11 (91.7)	1 (50.0)	11 (78.6)	0.637
OR admission (%)	1 (6.2)	2 (5.6)	0 (0.0)	0 (0.0)	0 (0.0)	0 (0.0)	0 (0.0)	0.941
APACHE II score	21.88 (10.11)	25.33 (9.62)	25.64 (6.44)	25.81 (9.57)	31.91 (10.36)	19.00 (2.83)	30.00 (6.60)	0.043
Shock (%)	5 (20.0)	53 (75.7)	9 (64.3)	15 (57.7)	11 (100.0)	0 (0.0)	9 (64.3)	<0.001
Immuno-compromised (%)	7 (28.0)	19 (27.1)	5 (35.7)	5 (19.2)	1 (8.3)	2 (100.0)	8 (57.1)	0.025
On IV antibiotics at study enrollment (%)	19 (76.0)	70 (100.0)	14 (100.0)	25 (96.2)	12 (100.0)	2 (100.0)	14 (100.0)	<0.001
Mortality at 60 days (%)	5 (20.0)	12 (17.1)	1 (7.1)	6 (23.1)	3 (25.0)	1 (50.0)	7 (50.0)	0.11
Procalcitonin (ng/ml - Tricore)	1.74 (2.96)	25.49 (29.65)	5.75 (15.75)	8.08 (20.79)	24.87 (42.88)	2.15 (1.27)	21.40 (29.26)	0.002
C-reactive protein (mg/dl - Tricore)	10.11 (6.48)	15.20 (4.70)	11.13 (6.33)	10.76 (6.38)	13.73 (4.20)	11.15 (0.35)	16.50 (4.27)	<0.001

ED, emergency department; OR, operating room; IV, intravenous.

For many diagnostics, considering multiple thresholds improves clinical actionability (e.g., procalcitonin thresholds of 0.1, 0.25, and 0.5 ng/ml). Consequently, we evaluated test characteristics for IMX-BVN-1’s predicted probabilities split into quartiles (Supplementary Table 4). The bacterial lowest quartile for LOSO CV, Stanford ICU, and Stanford ICU <36 h subgroup showed negative likelihood ratios of 0.055, 0.16, and 0.035, respectively, and upper quartile positive likelihood ratios of 40, 7.24, and 10, respectively. These values translate to lower-quartile sensitivities of 0.97, 0.91, and 0.98, and upper quartile specificities of 0.99, 0.95, and 0.96, for the LOSO CV, Stanford ICU, and Stanford ICU <36 h subgroup, respectively.

### IMX-BVN-1 compared with standard clinical biomarkers

We compared performance of IMX-BVN-1 to that of PCT and CRP only for microbiology-positive patients, since PCT was used by the adjudicators in determining bacterial infection status in microbiology-negative cases. In the 93 patients with available PCT and CRP, the bacterial-vs.-other AUROCs of PCT, CRP, and IMX-BVN-1 were 0.83 (95% CI 0.75–0.92), 0.70 (95% CI 0.6–0.81), and 0.87 (95% CI 0.8–0.94), respectively **(**Supplementary Fig. 8**)**. The viral-vs.-other AUROC of PCT, CRP, and IMX-BVN-1 were 0.27 (95% CI 0.14–0.39), 0.38 (95% CI, 0.23–0.53) and 0.86 (95% CI 0.74–0.99), respectively. The very low performance of CRP and PCT in separating viral from non-viral causes is expected but shows a clinically important aspect of IMX-BVN-1. Further, CRP and PCT have different courses of concentration in patients. Examining these 93 patients using typical PCT thresholds and the IMX-BVN-1 quartiles (Tables 3 and 4), the vast majority of noninfected cases have a PCT > 0.5 ng/ml. Thus, despite a reasonable AUROC, PCT would be of little clinical utility in this cohort because most noninfected patients are still above the highest cutoff for bacterial infections. In contrast, IMX-BVN-1 shows increasing probabilities of infection across quartiles for both bacterial and viral scores.

**Table 3:** Bacterial diagnosis by thresholds.

	Non-bacterial	Bacterial	Percent bacterial in band
IMX-BVN-1			
Quartile 1 (lowest)	21	4	16%
Quartile 2	12	8	40%
Quartile 3	2	23	92%
Quartile 4 (highest)	2	21	91%
Procalcitonin (ng/ml)			
<0.1	3	2	40%
0.1–0.25	11	1	8%
0.25–0.5	7	3	30%
>0.5	16	50	70%

Numbers of patients split into predicted probability quartiles for IMX-BVN-1 and split per pre-set thresholds for PCT for microbiology-confirmed Stanford ICU cases that had both scores available (*N* = 93).

**Table 4:** Viral diagnosis by thresholds.

	Non-viral	Viral	Percent viral in band
IMX-BVN-1			
Quartile 1 (lowest)	20	0	0%
Quartile 2	23	1	4%
Quartile 3	23	3	12%
Quartile 4 (highest)	13	10	43%
Procalcitonin (ng/ml)			
<0.1	3	2	40%
0.1–0.25	8	4	33%
0.25–0.5	8	2	20%
>0.5	60	6	9%

Numbers of patients split into predicted probability quartiles for IMX-BVN-1 and split per pre-set thresholds for PCT for microbiology-confirmed Stanford ICU cases that had both scores available (*N* = 93).

### Uncertain and mixed infection status

**Fig. 3:**
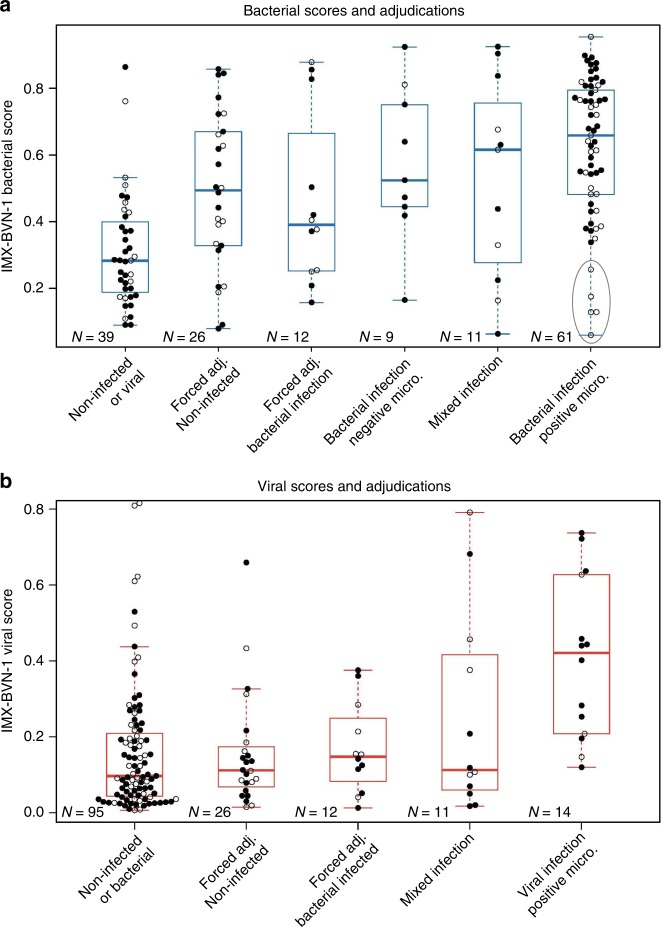
IMX-BVN-1 predicted probabilities in the Stanford ICU cohort across all clinical adjudication outcomes for bacterial and viral infections. The gray oval in (**a**) highlights a small number of patients with low bacterial scores, all of whom received antibiotics and were admitted >36 h prior to enrollment. *X*-axis categories indicate adjudicated infection status. Open circles indicate admission timing (black/closed ≤ 36 h, white/open >36 h). For each boxplot, the box shows the median and 25^th^–75^th^ quartile range (IQR), and the whiskers extend to the most extreme data point no further from the box than 1.5 times the IQR. Adj., adjudication; Micro., microbiology.

Patients with post hoc non-unanimous adjudications are of great interest. However, their assigned labels also have a high chance of being incorrect: only one physician needs to change his or her mind to have the class switch from noninfected (i.e. 1/3 votes infected) to infected (i.e. 2/3 votes infected). We plotted IMX-BVN-1 scores across all adjudication levels (Fig. 3). Generally, IMX-BVN-1 infection scores rise with increasing certainty of infection, though some bacterial-infection patients enrolled >36 h after hospital admission and already on antibiotics have low IMX-BVN-1 bacterial scores. Still, it is unclear whether this effect arises from disease progression over time or is due to time on IV antibiotics; IMX-BVN-1 scores are substantially higher in patients with <24 h of antibiotics treatment prior to enrollment (Supplementary Table 5).

Clinical descriptions of mixed-infection patients along with IMX-BVN-1, PCT, and CRP scores are in Supplementary Table 6. Notably, 5/7 samples with bacterial-viral coinfections in the <36 h subgroup are in the top two quartiles of IMX-BVN-1 bacterial score.

## Discussion

Despite intense study in multi-mRNA host-response diagnostics for acute infections and sepsis over the past two decades^15,24–38^, no multi-mRNA panel combined with a machine learning algorithm has been successfully applied with fixed weights in external data, much less translated to clinical application. Here, we show that the Inflammatix Bacterial-Viral-Noninfected (IMX-BVN-1) classifier has high accuracy in an independent cohort profiled using an entirely different technology. We also demonstrated advantages over standard-of-care diagnostics such as PCT and CRP. We overcame several challenges in successfully doing so, including (1) accounting for and leveraging substantial clinical, biological, and technical heterogeneity across multiple independent cohorts; (2) transferring a fixed-weight model learned using microarray data to a new diagnostic platform; and (3) learning from relatively small training data.

In order to be successfully translated to the clinic, a novel diagnostic must account for the substantial heterogeneity of the real-world patient population^43^. IMX-BVN-1 showed excellent, clinically relevant performance in diagnosing both bacterial and viral infections across 19 separate clinical studies composed of more than 1,100 patient samples. This performance arose from several factors, including: (1) training data from multiple clinical settings that collectively better represent real-world patient population, (2) reduced bias in training due to LOSO CV, (3) feature transformation (geometric mean scores) and (4) characteristics of the MLP. We also note that the Stanford ICU validation dataset was clinically quite distinct from our training data, which further demonstrates the generalizability of our results. Furthermore, compared to PCT and CRP, IMX-BVN-1 showed both an improved stratification of bacterial infections in the Stanford ICU setting while also identifying viral infections with high accuracy. We plan to ultimately present both a “bacterial-vs.-other” and a “viral-vs.-other” score to clinicians in tandem. This would allow physicians to both rule in and rule out bacterial and viral infections simultaneously, providing a combination of capabilities missing from the current clinical toolset. We also note that we have demonstrated the general training schema, architecture, and performance of the first version of our IMX-BVN classifier. We anticipate that future versions of the model will substantially improve with training on additional data and architecture enhancements. In contrast, the performance of PCT and other single-analyte biomarkers are fixed and cannot improve over time.

Determining which patients have bacterial infections is a daily challenge across many healthcare practices. The choice of whether to prescribe empiric antibiotics is essentially an educated guess, but one that carries serious risk of morbidity and mortality. Thus, the stringent test characteristics of IMX-BVN-1 for some patients (i.e., sensitivity of 97% and specificity of 99% for bacterial infections in the bottom and top quartiles, respectively) is potentially of high utility. However, such bands need not be based on quartiles and can be further calibrated to balance actionability with the number of patients in the band.

Novel diagnostic tools may have the greatest impact in patients for which there is greatest equipoise/uncertainty^7^. When a clinician is uncertain about the infection status of a patient (either 1 or 2 votes for an infection), the IMX-BVN-1 algorithm often assigns very high or low infection scores, as opposed to only “intermediate” scores **(**Fig. 3**)**. We do not know whether IMX-BVN-1 is correct in these cases, but it certainly deserves further study. We further note that the certainty of adjudication here comes only with extensive retrospective chart review; at hospital admission, there is very often high diagnostic uncertainty regarding presence and type of infection. To wit, in this cohort, 76% of consensus-noninfected patients, 96% of forced-adjudicated-noninfected patients, and 100% of viral-infected patients were on IV antibiotics at study enrollment.

Five late-enrolled, microbiology-positive samples had low bacterial scores in IMX-BVN-1 (Fig. 3—oval). It is possible that these samples had a resolution of their immune response due to antibiotics, and so their IMX-BVN-1 scores fell by the time of sampling. This pattern is consistent with previous results that showed reductions in gene expression score following antibiotic treatment^11^. Whether such patients would have had higher scores at admission, and how IMX-BVN-1 responds longitudinally to antibiotic treatment, requires further study.

IMX-BVN-1 is a MLP with hidden layers with linear activations, trained using gradient descent with mini-batches. Arguably, such an architecture could be mathematically represented as multinomial logistic regression, but multinomial logistic regression trained using least-squares did not reach nearly the same level of performance as IMX-BVN-1. These findings indicate that the model training and selection procedures played an important role in the discovery of IMX-BVN-1 and will be the subject of future work. In addition, our finding of improved stability using geometric mean scores computed from the input mRNAs suggests further research should focus on other feature transformations, either applied prior to learning or learned as part of a more general neural-network architecture.

We investigated methods for training machine learning models for diagnosing acute infections across multiple heterogeneous studies. Our results demonstrate that, in this domain, k-fold CV produces substantially biased estimates of performance. In fact, in some cases the top k-fold CV models performed worse than random on outer-fold HiCV test data. In conventional k-fold CV, random partitioning of the training data would likely result in the appearance of samples from the same study in both the learning folds and the corresponding left-out fold (in a way, a contamination of training data with external validation/test samples), leading to higher CV performance estimates. One is implicitly making the assumption that unseen samples are very similar to samples seen in the training data. Our results demonstrate that this assumption does not apply when the clinical population of interest is not well-represented in the training dataset and/or when the clinical population is sufficiently heterogeneous. We showed that the LOSO CV approach is more effective in identifying generalizable models in this domain.

Our work has several limitations. First, the Stanford ICU validation dataset is relatively small compared to the IMX training data. Thus, the confidence intervals are much wider on the validation cohort, pointing to the need for further validation. Second, we excluded patients with two or more types of positive microbiology from analysis, owing to uncertain adjudication. Third, test samples were assayed by NanoString, which may not be fast enough for most clinical applications. However, we are developing a rapid version of IMX-BVN-1, called “HostDx™ Sepsis”, on a purpose-built diagnostic instrument. Fourth, while IMX-BVN-1 shows great promise, the model was specifically tuned to our previously identified set of 29 markers. As such, the generalizability of our machine learning methodology has not been established for other diseases or other gene sets within acute infection. Finally, we restricted our analyses to adults; inclusion of pediatric samples in training and validation will be part of future studies. In general, further clinical studies with larger sample sizes are needed to confirm the diagnostic performance of IMX-BVN-1 in multiple clinical settings. Furthermore, as more “validation” studies are completed, we may be able to add them into “training” studies for future versions of IMX-BVN.

Overall, our research demonstrates the feasibility of successfully learning accurate, generalizable classifiers for acute bacterial and viral infections (or sepsis) using multiple heterogeneous training datasets. We further show that we can maintain high accuracy when applying the classifier, without retraining, to a new diagnostic platform. This work provides a potential roadmap for more general molecular diagnostic classifier development in other fields by leveraging vast repositories of transcriptomic data in concert with robust machine learning.

## Methods

### Systematic search for training studies

We compiled the training dataset (“IMX”; Inflammatix) by identifying studies from the NCBI GEO and EMBL-EBI ArrayExpress databases using a systematic search^12,22^. For included studies, patients (1) had to be physician-adjudicated for the presence and type of infection (i.e. bacterial infection, viral infection, or non-infectious inflammation), (2) were at least 18 years of age, (3) had been seen in hospital settings (e.g. emergency department, intensive care), (4) had either community- or hospital-acquired infection, and (5) had blood samples taken within 24 h of initial suspicion of infection and/or sepsis. In addition, each study had to measure all 29 host mRNAs of interest and have at least five healthy samples. Included studies were individually normalized from raw data (Supplementary Methods), and then co-normalized using COCONUT^12^.

### Iterative COCONUT normalization for platform matching

Diagnostic development in microarrays has typically suffered from a “last-mile” problem of clinical translation: a classifier trained only on gene expression data from microarrays would not be directly applicable on a rapid clinical diagnostic platform due to differing measurement types. We hypothesized that a dataset from one technical background (e.g., microarrays) could be “matched” to another technical background (NanoString nCounter targeted mRNA quantitation) through an iterative application of COCONUT, allowing a classifier trained using microarrays to be directly applied in samples profiled on the NanoString platform, without the need to train using NanoString data.

We measured the 29 target mRNAs in a set of whole-blood samples from 40 healthy controls collected in PAXgene RNA tubes and taken across four different sites in the USA. We acquired the healthy control samples commercially (10 samples through Biological Specialties Corporation, Colmar, PA USA; 30 samples through BioIVT Corporation, Hicksville, NY USA). Donors self-reported as healthy and received negative test results for both HIV and hepatitis C. They were not age-matched or sex-matched to either the training or validation data (further details in Supplement). We then iteratively applied the COCONUT algorithm^12^, adjusting the means and variances of the distributions of expression for the 29 target mRNAs in healthy control samples of the IMX microarray studies to align with their corresponding distributions in the commercial healthy controls assayed by NanoString (Supplementary Methods; Supplementary Fig. 2). These adjustments were then applied to the expression values of all samples in IMX to enable application of the trained classifier on the Stanford ICU samples.

### mRNA feature sets

Our analysis included 29 mRNA targets (listed in the Supplement) composed of three separate, validated sub-panels: the 11-mRNA “Sepsis MetaScore”^11^, 7-mRNA “Bacterial-Viral MetaScore”^12^, and 11-mRNA “Stanford mortality score”^13^. Each score is composed of two geometric mean (GM) modules. We explored two methods for developing a classifier using these 29 mRNAs as input features: (1) using the 29 expression values without applying any transformations, and (2) using GMs of the six original modules as input features to the classifier.

### Model selection and hyperparameters

We evaluated four types of classification models: (1) LR with a lasso (L_1_) penalty, (2) SVM with radial basis function kernel, (3) XGBoost and (4) MLPs, a type of feed-forward neural network. We chose these models because of their prior use in other diagnostic applications and their ability to accommodate multiclass classification. Hyperparameter search procedures for each model are described in Supplementary Methods.

### Classifier evaluation metrics

The AUROC is a common metric to evaluate binary classifiers but there is not a widely adopted generalization for a multiclass problem such as ours (bacterial vs. viral vs. noninfected). We selected our best classifier based on the APA, defined as the mean of the three one-class-versus-all AUROCs (i.e., the bacterial-vs.-other, viral-vs.-other, and noninfected-vs.-other AUROCs), which allowed us to rank models across all three classes. However, as the bacterial-vs.-other and viral-vs.-other AUROCs are arguably more relevant metrics to clinical practice, we also report these individual measures of performance for our final classifier. In IMX, we computed 95% confidence intervals for AUROCs based on 5000 bootstrap samples of a given classifier’s predicted probabilities. In the Stanford ICU dataset, we computed AUROC confidence intervals using the method of Hanley and McNeil due to small sample size.

### Methodological evaluation of cross-validation and input features

Our final classifier development depended on choices of (1) the CV method used for model selection and (2) the type of input features used for classifier training. We considered two types of CV strategies (traditional k-fold vs. LOSO) and two types of input features (6-GM scores vs. 29-mRNA expression values) for classifier training (Supplementary Methods). To decide which combination of CV strategy and input features to use for our classifier development, we performed a third type of cross-validation called hierarchical CV (HiCV). HiCV simulates the process of machine learning classifier development and independent testing by partitioning training data into pairs of (inner, outer) folds (Fig. 1a), and is reliable in small sample size scenarios^45,46^. Each inner fold is used to simulate the entire modeling process (i.e. hyperparameter search via CV followed by training of the best selected model). The corresponding outer fold is then used to test each classifier. The process is repeated for each inner/outer fold pairing. To draw conclusions robust to variability in how the data were partitioned, we split the IMX data into three folds for HiCV.

We based our decision of which CV method and input feature type to use in classifier development on both the performance on the outer fold (i.e. performance in “external validation”) and the bias (i.e. difference between inner fold CV and outer fold performance). We hypothesized that modeling choices resulting in higher outer-fold performance and lower bias may be more likely to produce generalizable models in formal classifier development.

### Stanford ICU Biobank

We collected blood into PAXgene RNA tubes from 163 patients enrolled in the Stanford University Medical ICU Biobank from 2015-2018 after written informed consent (Stanford IRB approval #28205). Adult subjects enriched for acute respiratory distress syndrome risk factors (e.g. sepsis, aspiration, trauma) were recruited at admission to the Stanford ICU from either the hospital wards or the emergency department as part of an existing biobanking study. Patients eligible for inclusion were consecutive adults (> = 18 years) admitted to Stanford ICU with at least one ARDS risk factor (e.g. sepsis, pneumonia, trauma, aspiration). We excluded routine post-op patients, those admitted for a primary neurologic indication, and those with anemia (hemoglobin <8). Screening of consecutive new admissions via electronic medical records review of all ICU subjects was performed by a study coordinator and the study PI (AJR). Screening occurred on weekdays with a goal enrollment in <24 h of admission to ICU, and included patients admitted to the ICU from the wards or the emergency room. Patients or their surrogates were approached for consent to participate in the Stanford ICU biobank, and the PAXgene tubes used for this study were collected between October 2015 to April 2017. We excluded two samples from analysis; one was collected >72 h after ICU admission, and one was excluded due to incomplete phenotype data at the time of mRNA analysis. Clinical samples were shipped frozen to Inflammatix, total RNA was isolated, and NanoString analysis performed by technicians blinded to clinical outcomes (Supplementary Methods). Our final classifier trained on the IMX dataset was then directly applied to the NanoString data, without any additional training.

Infection status was adjudicated by three physicians who had access to the entire electronic medical record for the admission (including physician notes, imaging, final culture data from blood and other specimens, clinical procalcitonin when drawn, start time and duration of antibiotics, and discharge summary). Each case was adjudicated as (1) Infected, (2) Probable infection, (3) Uncertain infection, (4) Not infected. All subjects were classified as noninfected, bacterial, viral, fungal, or mixed infection, and each vote for the presence of infection was weighted equally. Every case that was adjudicated as probable or possible infection was reviewed by three physicians (AJR, BS, ARM) using all electronic medical record data as above. Probable infections were all culture negative but adjudicated unanimously as infected by all three physicians. The uncertain cases underwent forced adjudication, with 0/3 or 1/3 voting for infection deemed “uninfected” and 2/3 or 3/3 voting for infection deemed “infected”. Across three reviewers, this yielded four classes for each infection type, from zero to three votes for the presence of an infection. Adjudicators were blinded to the gene expression data. Clinical procalcitonin data within 24 h of admission was available for ~1/4 of the cohort and may have influenced both clinical treatment (e.g. antibiotic duration) and adjudication of infection status. Mixed infections were not included in the main performance analyses.

### Reporting summary

Further information on research design is available in the Nature Research Reporting Summary linked to this article.

## Supplementary information

Supplementary Information

Description of Additional Supplementary Files

Supplementary Data 1

Reporting Summary

## Data Availability

Gene expression data are publicly available at their stated accession IDs (Table 1). The normalized NanoString data is available as Supplementary Data 1.

## Code availability

The core software used in developing IMX-BVN-1 is described in the Supplementary Methods, including open source software tools. Further details are available on request. The proprietary portions of the code are owned by Inflammatix and are not available.
