# Supplementary material for: A generalizable 29-mRNA neural-network classifier for acute bacterial and viral infections
Source: Nat Commun. 2020 Mar 4;11:1177. doi: 10.1038/s41467-020-14975-w (PMC7055276; doi:10.1038/s41467-020-14975-w)

Supplementary Information for:

A generalizable 29-mRNA neural-network classifier for acute bacterial and viral infections

Michael B Mayhew[1], Ljubomir Buturovic[1], Roland Luethy[1], Uros Midic[1], Andrew R Moore[2], Jonasel Roque[3], Brian Shaller[3], Tola Asuni[3], David Rawling[1], Melissa Remmel[1], Kirindi Choi[1], James Wacker[1], Purvesh Khatri[4,5], Angela J Rogers[3], Timothy E Sweeney[1]*

Affiliations:
1- Inflammatix, Inc., 863 Mitten Rd, Suite 104, Burlingame, CA, 94010, USA
2- Department of Medicine, Stanford University, Palo Alto, CA 94305, USA
3- Division of Pulmonary, Allergy, and Critical Care Medicine, Department of Medicine, Stanford University, Palo Alto, CA 94305, USA
4- Institute for Immunity, Transplantation and Infections, Stanford University, Palo Alto, CA 94305, USA
5- Center for Biomedical Informatics Research, Department of Medicine, Stanford University, Palo Alto, CA 94305, USA

Table of contents

Supplementary Methods

Supplementary Tables 1-6

Supplementary Figures 1-8

Supplementary Methods

Normalization and COCONUT co-normalization of expression data

We first performed normalization within each study, adopting one of two approaches depending on the platform. For Affymetrix arrays, we normalized the expression data using either RMA or gcRMA. For other platforms, we normalized expression data using the normal-exponential convolution approach for background correction followed by quantile normalization.

Following normalization of the raw expression data, we used the COCONUT algorithm to co-normalize these measurements and ensure that they were comparable across studies. COCONUT builds on the ComBat empiric-Bayes batch correction method, computing the expected expression value of each gene from healthy patients and adjusting for study-specific modifications of location (mean) and scale (standard deviation) in the gene's expression. For our analyses, we use the parametric prior of ComBat in which gene expression distributions are assumed to be Gaussian and the empirical prior distributions for study-specific location and variance modification parameters are Gaussian and Inverse-Gamma, respectively.

Machine learning terminology

In our manuscript, *model* refers to a type of machine learning algorithm, such as logistic regression, decision tree, neural network, etc. *Classifier* refers to a model with fixed parameters, ready to be applied to previously unseen samples. Classifiers use two types of parameters: weights, which are learned by the core algorithm during training (such as stochastic gradient descent), and, for some models, 'hyperparameters' which control the training procedure and configuration of the model and are set prior to training. In classifier development, weights are learned with a given set of hyperparameters on given training data.

K-fold vs LOSO CV

A conventional approach to develop classifiers capable of generalizing well to unseen samples is cross-validation (CV). We considered two different types of CV schemes: k-fold CV and leave-one-study-out (LOSO) CV. In k-fold CV, we randomly partitioned all samples into k =5 non-overlapping folds of roughly similar sample sizes. An underlying assumption of k-fold CV is that the cross-validation training and validation samples are drawn from the same distribution. In LOSO CV, one entire study is left out as each 'fold'. LOSO CV is more conceptually aligned to clinical application of diagnostic classifiers in heterogeneous clinical settings, and non-machine-learning-based multi-gene diagnostic panels selected using LOSO have been shown to be highly generalizable[13,18]. In both approaches (k-fold and LOSO),

candidate classifiers are trained on all folds but one, and then tested in the left-out fold, and the procedure is repeated once for each fold. We pooled the predicted probabilities across all left-out folds and computed performance metrics on the pooled predictions. We note that left-out studies in LOSO CV vary in size, with some studies having quite small sample sizes. By pooling predicted probabilities and computing a model's overall performance based on these pooled predictions (rather than computing metrics for each left-out study and averaging the metrics across studies), we account for differences in study size and ensure that models with high overall performance generate consistent predictions across studies.

mRNA feature sets

We here used only the mRNA targets from previously defined diagnostic gene scores[1-3]. Each of these three previously-described scores is calculated as the difference in geometric means (GMs) of the expression values of two gene 'modules' (one composed of over-expressed genes and the other composed of under-expressed genes). The modules are: (1) infection-up: *CEACAM1, ZDHHC19, C9orf95, GNA15, BATF, C3AR1;* (2) infection-down: *KIAA1370, TGFBI, MTCH1, RPGRIP1, HLA-DPB1*; (3) bacterial-viral-up: *HK3, TNIP1, GPAA1, CTSB*; (4) bacterial-viral-down: *IFI27, JUP, LAX1*; (5) mortality-up: *DEFA4, CD163, RGS1, PER1, HIF1A, SEPP1, C11orf74, CIT*; and (6) mortality-down*: LY86, TST, KCNJ2.* One mRNA target, OR52R1, was removed from the panel because it has no introns, preventing assay development.

Model hyperparameter search

For the four types of classifiers we consider, hyperparameters must be selectively searched (e.g. by random sampling) to optimize classifier performance. LR has one hyperparameter, the lasso penalty coefficient, while SVM has two hyperparameters, the C penalty term and the kernel bandwidth, γ. For these two models, we performed grid search. In both HiCV and LOSO CV with the full IMX dataset, LR was trained based on a grid search of 200 values of the lasso penalty coefficient while SVM was trained using a grid search of 100 cost values and 100 values of the bandwidth parameter for a total of 10,000 (C, γ) value pairs.

For XGBoost and MLP, we observed significant variability due to pseudo-random initialization, and chose to include the pseudo-random-number generator seed among hyperparameters. For XGBoost, we sampled randomly values of the following hyperparameters: (1) seed, (2) learning rate, (3) minimum loss reduction required to introduce a split in the classifier tree, (4) maximum tree depth, (5) minimum child weight, (6) minimum sum of instance weights required in each child, (7) maximum delta step, (8) L_2 penalty

coefficient for weight regularization, 9) tree method (exact or approximate), and 10) number of training rounds[4].

For MLP, we fixed the mini-batch size to 128 (we had not observed any effect of mini-batch size on model performance in preliminary analyses) and the optimization algorithm to ADAM[5]. We chose ADAM as our model optimizer for two main reasons: 1) it has been widely adopted in the deep learning literature, 2) ADAM demonstrates attractive and fast convergence properties based in large part on its adaptive estimation of learning rates for each model parameter. We randomly sampled values of the following hyperparameters for MLP: (1) number of hidden layers, (2) number of nodes per hidden layer, (3) type of activation function for all hidden layers, (4) learning rate, (5) number of training iterations, (6) type of weight regularization, (7) seed, and (8) presence and extent of dropout for the input and hidden layers. We fixed the β_1, β_2, and ε parameters of ADAM to 0.9, 0.999, and 1e-08 respectively. In HiCV, XGBoost was trained with 10,000 randomly sampled hyperparameter configurations; in LOSO CV for final classifier development, it was evaluated with 30,000 randomly sampled hyperparameter configurations. In HiCV, MLP was evaluated with 100,000 hyperparameter configurations, randomly sampled from grids for some hyperparameters and continuous value ranges for others[4]. In LOSO CV for final classifier development, MLP hyperparameters were searched using the fine-tuning procedure described below.

MLP performance was highly sensitive to the value of the random seed used for initialization of the network's parameters (e.g. weights and biases) as well as other aspects of the network training procedure. To mitigate this effect and identify promising points in the space of network weights and biases, we first defined a large grid of hyperparameters, excluding the seed. We then evaluated each hyperparameter combination from the grid in combination with 250 seed values. Upon completion of this initial search, we focused on the most promising hyperparameter combinations. We sampled a new set of 750 seed values and then evaluated them in combination with the most promising hyperparameter configurations of the initial search.

For each hyperparameter configuration, we pooled predicted probabilities from the left-out studies, and calculated APA with the pooled probabilities. Each classifier was thus trained and evaluated on all samples in the IMX dataset. The configuration with the highest APA was selected as the final winning set of hyperparameter values.

Hyperparameter searches for both LR and SVM models were performed with local computing resources (i.e. personal laptops), generally finishing in a few hours. Hyperparameter searches for XGBoost models were also performed on personal laptops. However, owing to the relatively higher dimensional hyperparameter space, XGBoost searches required one or two days to complete. MLP hyperparameter searches for HiCV were conducted using 40 Amazon AWS c4.8xlarge instances and completed within 8-

12 hours. We performed the fine-tuning procedure used to identify promising seeds as well as hyperparameters for the MLP on our personal laptops, with searches finishing after no more than two days.

Iterative COCONUT matching pseudocode

In order to make a version of microarray data on which we could train a model that would be directly applicable in the NanoString platform, we iteratively applied the COCONUT co-normalization algorithm. In the iterative COCONUT procedure, the commercial healthy samples represent the *target* dataset as they remain unchanged over the course of the iterative procedure, and the IMX healthy samples represent the *query* dataset we wish to make similar to the *target* dataset. This procedure terminated when the mean absolute deviation (MAD) between the vectors of average expression of the 29 diagnostic markers in both the IMX and commercial healthy datasets did not change by more than 0.001 in consecutive iterations. After iterative application of COCONUT, we obtained some values <1 in the training datasets; these were truncated to 1 as this is the minimum NanoString value possible. Pseudocode for this procedure is:

1) *Run COCONUT to co-normalize query studies to one another to get dataset Q*
2) *Set* δ_{last} *= 0.0*
3) *while* $\tau > 0.001$
   a) *Run COCONUT to co-normalize Q and target dataset, T, to get* Q_{upd} *and* T_{upd}
   b) *Set* $Q = Q_{upd}$
   c) *Compute* $\mu_{query} = \frac{\sum_{i=1}^{n_{query}} q_i}{n_{query}}$ *where* q_i *is a row vector from Q of 29-marker expression values for sample i*
   d) *Compute* $\mu_{target} = \frac{\sum_{j=1}^{n_{target}} t_j}{n_{target}}$ *where* t_j *is a row vector from T of 29-marker expression values for sample j*
   e) *Compute* $\delta_{cur} = \frac{\sum_{k=1}^{29} |\mu_{query} - \mu_{target}|}{29}$
   f) *Compute* $\tau = \delta_{cur} - \delta_{last}$
   g) *Set* $\delta_{last} = \delta_{cur}$

Description of healthy control samples used for NanoString expression alignment

Prospectively collected healthy control samples were obtained commercially from two vendors across multiple collection sites. To be deemed "healthy," patients were non-febrile with no symptoms of

infection or other known illness on the day of and for at least 3 days prior to collection and were not undergoing antibiotic treatment. Samples were collected in PAXgene Blood RNA tubes per the manufacturer's protocol, frozen at -80°C, and shipped frozen on dry ice. All samples were tested with FDA CBER licensed screening tests to show the following:

- Hepatitis B Surface Antigen Negative
- HBV NAT Negative
- NIV 1&2 Antibody Negative
- HIV NAT Negative
- HCV Antibody Negative
- HCV NAT Negative
- Syphilis Negative
- West Nile Virus NAT Negative
- HTLV I/II Negative
- T. Cruzi Antibody Negative

10 samples were obtained through Biological Specialties Corporation (Colmar, PA). 30 samples were obtained through BioIVT Corporation (Hicksville, NY).

Software implementation details

Data pre-processing and normalization were performed in the R programming language (https://www.r-project.org/) while NanoString sample normalization routines were implemented in Python (https://www.python.org/). Machine learning software and downstream analyses were also implemented in Python. Neural network development used a combination of native Tensorflow (https://www.tensorflow.org/) and Keras (https://keras.io/) deep learning frameworks. Analyses were carried out on a combination of local compute resources and Amazon Web Services EC2 instances.

Stanford ICU NanoString expression profiling

Clinical samples were shipped frozen to Inflammatix and run by technicians blinded to clinical outcomes. To generate NanoString expression for the Stanford ICU samples, we isolated RNA from PAXgene RNA tubes with the RNeasy Plus Micro Kit (Qiagen, part #74034) on a QIAcube (Qiagen), using

a custom protocol. Each NanoString expression profiling reaction consisted of 150ng of RNA per sample hybridized for 16 hours at 65° C per manufacturer's instructions. We then followed the nCounter SPRINT standard protocol to generate mRNA counts. We normalized the raw mRNA counts across samples using the geometric mean of counts for 4 housekeeping genes (CDIPT, KPNA6, RREB1, YWHAB), per manufacturer instructions.

Reference biomarkers

Commonly used biomarkers for infection diagnosis, such as procalcitonin and C-reactive protein (CRP), were run only at the treating physician's discretion, and were frequently missing. We thus measured procalcitonin and CRP from frozen serum at a CLIA/CAP-compliant reference laboratory (TriCore, Albuquerque, NM).

Supplementary References

1. Sweeney TE, Shidham A, Wong HR, Khatri P. A comprehensive time-course-based multicohort analysis of sepsis and sterile inflammation reveals a robust diagnostic gene set. *Sci Transl Med*. 2015;7(287):287ra271.
2. Sweeney TE, Wong HR, Khatri P. Robust classification of bacterial and viral infections via integrated host gene expression diagnostics. *Sci Transl Med*. 2016;8(346):346ra391.
3. Sweeney TE, Perumal TM, Henao R, et al. A community approach to mortality prediction in sepsis via gene expression analysis. *Nat Commun*. 2018;9(1):694.
4. Bergstra J, Bengio Y. Random Search for Hyper-Parameter Optimization. *Journal of Machine Learning Research* 2012;13:281-305.
5. Kingma D, Ba J. ADAM : A Method for Stochastic Optimization. *ArXiv*. 2017;arXiv:1412.6980v9.

Supplementary Tables

Supplementary Table 1. Assignment of studies to outer folds for hierarchical cross-validation (HiCV) analyses.

Outer fold 1:

STUDY	BACTERIAL	VIRAL	NONINFECTED
EMEXP3589	4	5	14
GSE13015 (GPL6106)	45	0	0
GSE20346	6	4	0
GSE40012	16	6	12
GSE60244	22	71	0
GSE65682	0	0	33
GSE69528	83	0	0
TOTAL	176	86	59
% OF TOTAL	0.55	0.27	0.18

Outer fold 2:

STUDY	BACTERIAL	VIRAL	NONINFECTED
EMTAB5273	228	0	0
GSE21802	0	12	0
GSE27131	0	7	0
GSE42834	14	0	68
GSE82050	0	24	0
GSE111368	0	33	0
TOTAL	242	76	68
% OF TOTAL	0.63	0.20	0.18

Outer fold 3:

STUDY	BACTERIAL	VIRAL	NONINFECTED
EMTAB1548	82	0	58

GSE13015 (GPL6947)	15	0	0
GSE28750	10	0	11
GSE57065	82	0	0
GSE68310	0	104	0
TOTAL	189	104	69
% OF TOTAL	0.52	0.29	0.19

Supplementary Table 2. Comparison of HiCV outer fold performance using GM scores vs. 29-mRNA inputs as features. Results shown are based on LOSO CV. Each column contains the average of APA values achieved by the top 50 models as ranked by LOSO CV in the inner fold. Compared within HiCV outer folds, the best 6-GM score models are uniformly noninferior to the best 29-mRNA models.

	HiCV outer fold 1		HiCV outer fold 2		HiCV outer fold 3	
	6-GM	29-mRNA	6-GM	29-mRNA	6-GM	29-mRNA
LR	0.75	0.75	0.83	0.80	0.75	0.69
SVM	0.78	0.74	0.87	0.75	0.66	0.60
XGBoost	0.78	0.78	0.80	0.76	0.67	0.66
MLP	0.74	0.66	0.75	0.75	0.72	0.67

Supplementary Table 3. IMX-BVN-1 AUROCs according to immunocompromised status. Immune compromise was defined mainly by presence of HIV/AIDS, s/p solid organ transplant, or recent cancer chemotherapy. Of the 109 defined-infection patients, 31 had immune compromise. Numbers in parentheses are 95% CI.

Stanford ICU single-infected cohort (N=109)

	Bacterial-vs.-other	Viral-vs.-other
Not immunocompromised	0.89 (0.82-0.96)	0.87 (0.72 – 1.0)
Immunocompromised	0.76 (0.6-0.93)	0.84 (0.61-1.0)

Stanford ICU <36h subgroup (N=70)

	Bacterial-vs.-other	Viral-vs.-other
Not immunocompromised	0.91 (0.83-0.99)	0.88 (0.69 – 1.0)
Immunocompromised	0.95 (0.82-1.0)	0.96 (0.83-1.0)

Supplementary Table 4. Test statistics per quartile for both bacterial-vs-other (A, C, E) and viral-vs-other (B, D, F) scores for IMX LOSO CV (A, B), Stanford ICU (C, D) and Stanford ICU <36h subgroup (E, F) cohorts. The lower two quartiles are treated as rule-out bands, and the upper two quartiles are treated as rule-in bands. LR: likelihood ratio.

While IMX-BVN-1 will be reported with four bands, not dichotomized, it is easy to calculate dichotomous results around any one of the presented quartiles by summing the remaining quartiles. For instance, by dichotomizing above the bottom quartile (to maximize sensitivity), the BVN-1 bacterial-vs-other test characteristics would be: IMX LOSO CV: 97% sens, 54% spec; Stanford ICU 91% sens, 54% spec, Stanford <36h subgroup, 98% sens, 65% spec (shown in last two columns of parts A, C and E, respectively).

A - IMX LOSO CV		Non-bacterial	Bacterial	LR	Treated as	Sensitivity in quartile	Specificity in quartile
Bacterial vs. other	Quartile 1 (lowest)	249	18	0.055	Rule out	0.970	
	Quartile 2	159	108	0.52	Rule out	0.822	
	Quartile 3	49	218	3.39	Rule in		0.894
	Quartile 4 (highest)	5	263	40.03	Rule in		0.989

B - IMX LOSO CV		Non-viral	Viral	LR	Treated as	Sensitivity in quartile	Specificity in quartile
Viral vs. other	Quartile 1 (lowest)	266	1	0.011	Rule out	0.996	
	Quartile 2	257	10	0.12	Rule out	0.962	
	Quartile 3	203	64	0.95	Rule in		0.747
	Quartile 4 (highest)	77	191	7.49	Rule in		0.904

C – Stanford ICU		Non-bacterial	Bacterial	LR	Treated as	Sensitivity in quartile	Specificity in quartile
Bacterial vs. other	Quartile 1 (lowest)	21	6	0.159	Rule out	0.914	
	Quartile 2	14	13	0.52	Rule out	0.814	
	Quartile 3	2	25	6.96	Rule in		0.949
	Quartile 4 (highest)	2	26	7.24	Rule in		0.949

D – Stanford ICU		Non-viral	Viral	LR	Treated as	Sensitivity in quartile	Specificity in quartile
	Quartile 1 (lowest)	27	0	0.000	Rule out	1.000	

**Viral vs. other**	Quartile 2	26	1	0.26	Rule out	0.929	
	Quartile 3	24	3	0.85	Rule in		0.747
	Quartile 4 (highest)	18	10	3.77	Rule in		0.811

**E - Stanford ICU <36h subgroup**		Non-bacterial	Bacterial	LR	Treated as	Sensitivity in quartile	Specificity in quartile
**Bacterial vs. other**	Quartile 1 (lowest)	17	1	0.035	Rule out	0.977	
	Quartile 2	8	9	0.66	Rule out	0.795	
	Quartile 3	0	17	Div/0	Rule in		1.000
	Quartile 4 (highest)	1	17	10.05	Rule in		0.962

**F - Stanford ICU <36h subgroup**		Non-viral	Viral	LR	Treated as	Sensitivity in quartile	Specificity in quartile
**Viral vs. other**	Quartile 1 (lowest)	18	0	0.000	Rule out	1.000	
	Quartile 2	17	0	0.00	Rule out	1.000	
	Quartile 3	15	2	0.72	Rule in		0.746
	Quartile 4 (highest)	9	9	5.36	Rule in		0.847

Supplementary Table 5. IMX-BVN-1 scores stratified by patients who had been on antibiotics <24h or were not on antibiotics (N=65) vs those on antibiotics for >=24 hours (N=44). Numbers in parentheses are 95% CI.

	Bacterial-vs.-other	Viral-vs.-other
No antibiotics or <24 hours	0.91 (0.84-0.98)	0.93 (0.8 – 1.0)
>=24 hours antibiotics	0.76 (0.61-0.9)	0.71 (0.47-0.96)

Supplementary Table 6. Vignettes and biomarker scores for patients with infection from mixed source (e.g. bacterial and viral).

Patient infection description	BVN1 bacterial score	BVN1 viral score	PCT - ng/ml	CRP - mg/dl
Mycoplasma hominis osteomyelitis ~1 week before developing pneumonia requiring transfer to ICU with broncheoalveolar lavage (BAL) with respiratory viral panel positive for Respiratory Syncytial Virus (RSV).	0.163	0.791	1.04	>19
Pneumonia with *Klebsiella pneumoniae* in sputum culture and RSV on respiratory viral panel. CT was read as arguing against viral pneumonia due to lack of ground glass opacities.	0.676	0.107	36.72	>19
Lung transplant patient with severe respiratory failure with Parainfluenza on respiratory viral panel. Also with elevated white blood cell count and procalcitonin, concerning for bacterial superinfection. Was on broad spectrum antibiotics, but difficult to interpret given medical complexity and immunosuppression	0.292	0.207	3.92	>19
Originally admitted with pneumonia with methicillin-resistant *Staphylococcus aureus* (MRSA) on BAL. subsequently found to have encephalitis with Herpes Simplex Virus and Varicella Zoster Virus in lumbar puncture. Transferred to ICU after aspiration event.	0.615	0.100	0.62	>19
Influenza positive with methicillin-sensitive *Staphylococcus aureus* (MSSA) pneumonia/bacteremia. Also with *Aspergillus fumigatus* and *Rhizopus* on respiratory cultures which was treated	0.925	0.017	>100	>19
Pneumonia with Influenza positive on respiratory viral panel, MSSA in sputum, and *Streptococcus pneumoniae* in sputum and on blood culture.	0.904	0.020	22.25	>19
Patient with Influenza A (H1N1) on respiratory viral panel and *Pasteurella multocida* bacteremia.	0.837	0.069	49.25	>19
Lung transplant patient with recent admission for Norovirus and *Clostridium difficile* colitis readmitted for respiratory failure and volume overload, not particularly septic. Cultures from chest wound with gas on CT grew *Acinetobacter baumannii* and sputum grew *Corynebacterium propinquum* in setting of new ground glass opacities on CT.	0.224	0.419	missing	missing
HIV positive male presented with sepsis. Found to have *Cryptococcus* on BAL and in cerebrospinal fluid. Some concern for bacterial infection throughout hospitalization, although no positive cultures, and was on broad spectrum antibiotics throughout.	0.445	0.376	9.8	12.6
Candida bacteriuria and likely spontaneous bacterial peritonitis (>250 PMNs at outside hospital paracentesis) as well as leukocytosis and elevated procalcitonin.	0.329	0.374	missing	missing

Influenza B and MSSA pneumonia	0.063	0.681	6.06	6.2
ARDS and septic shock due to Parainfluenza pneumonia. Patient also had elevated procalcitonin, concerning for possible bacterial superinfection	0.324	0.456	3.54	17.2
Shock with *Enterococcus faecalis* bacteremia. Also found to be RSV positive on respiratory viral panel.	0.438	0.118	21.9	10.8
Cough and respiratory symptoms with infiltrates on chest X-ray in setting of neutrophil count of 0. MSSA grew from BAL, however, additional concern for fungal pneumonia in setting of immunosuppression, given elevated galactomannan of 0.76 in BAL. Was treated empirically for both fungal and bacterial pneumonia.	0.630	0.169	1.72	18.2

Supplementary Figures

Supplementary Figure 1. Overall study schema. COCONUT – COmbat CONormalization Using conTrols; CV – cross-validation; LOSO – leave-one-study-out; HiCV – hierarchical cross-validation; LR – logistic regression; SVM – support vector machines; MLP – multi-layer perceptron; PCT – procalcitonin; CRP – C-reactive protein.

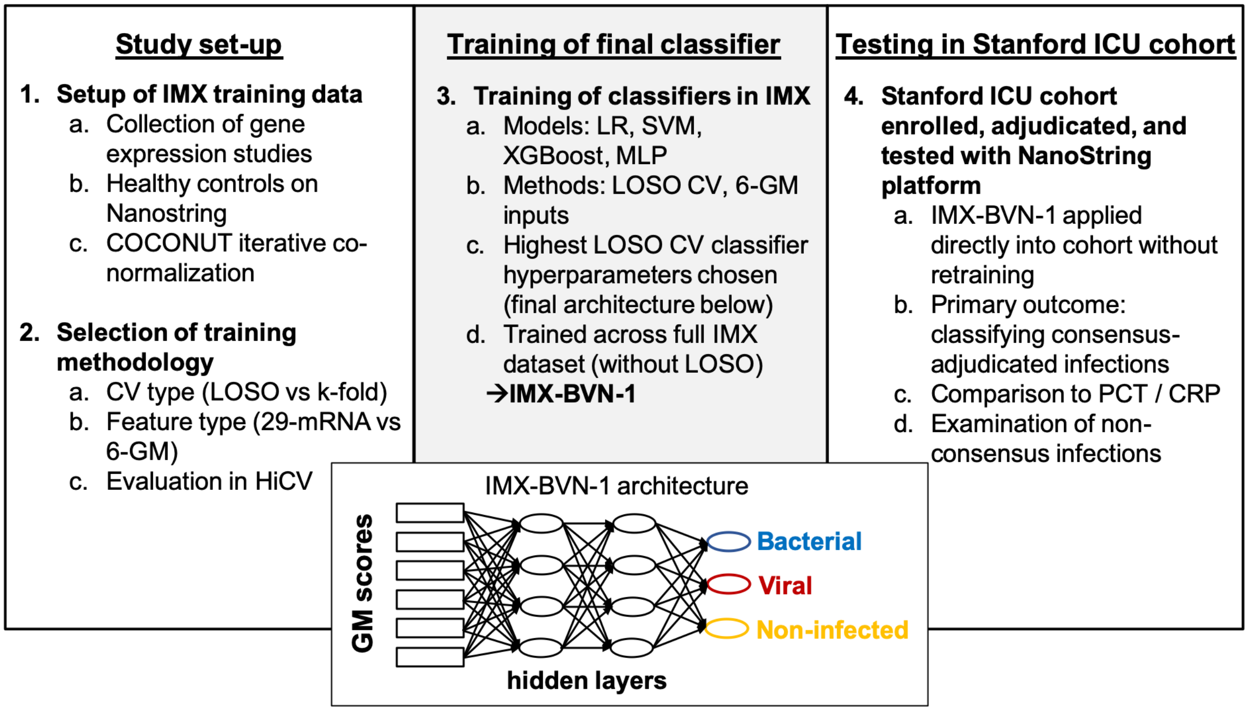

Supplementary Figure 2. Iterative COCONUT alignment. "Reference" = IMX data; "Target" = NanoString data. Shown are density plots of commercial healthy NanoString expression (blue) and IMX expression (pink), for all 29 diagnostic markers. The microarray distributions are shown at three distinct iterations in the co-normalization-based alignment process. Dashed lines indicate distributions of expression of the given reference gene at intermediate iterations (both the first iteration of the procedure and the iteration marking the halfway point of the procedure) while solid lines show the distribution at termination of the procedure. The distributions of the target and query datasets become visually closer over the course of the procedure, as expected.

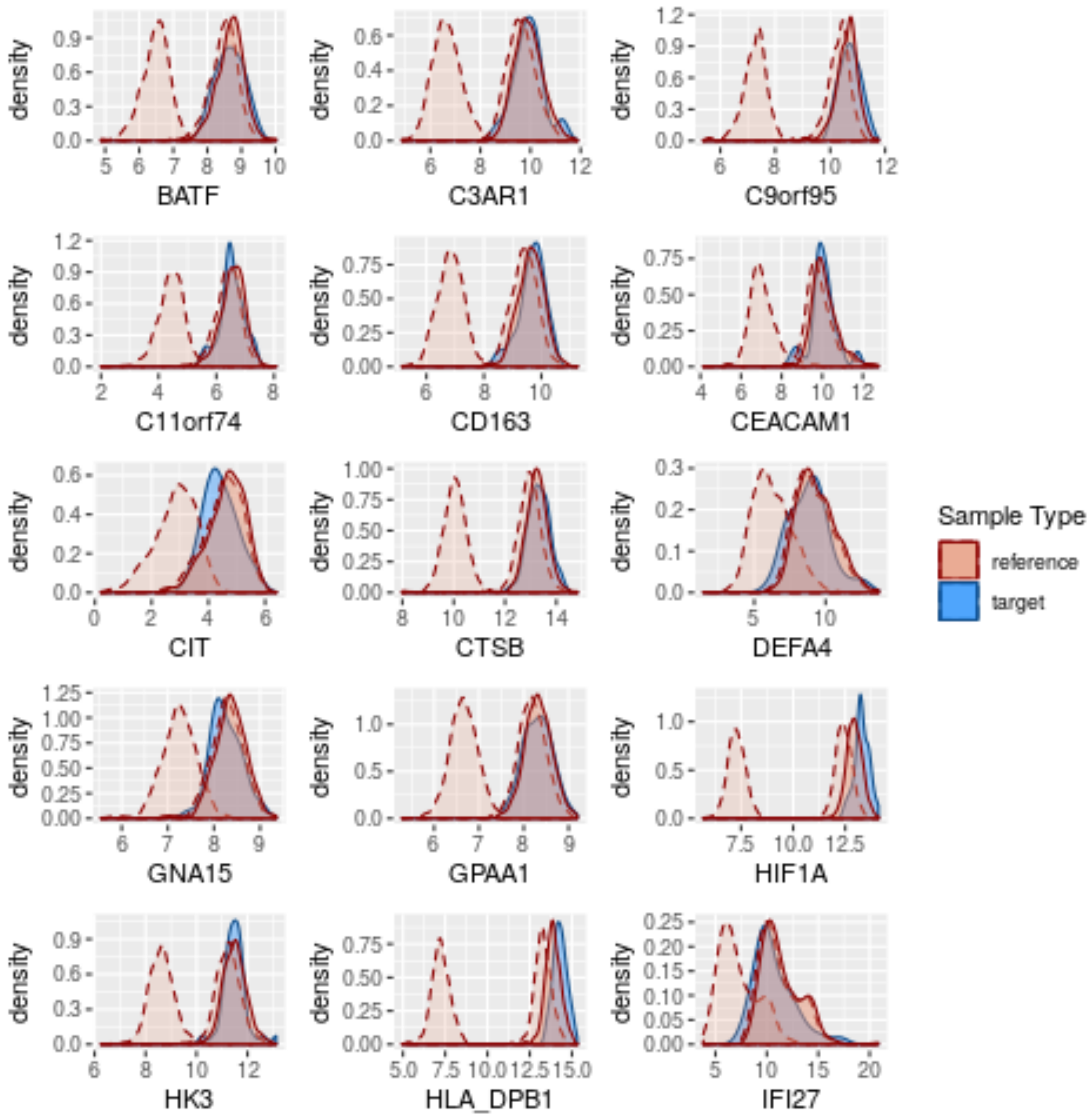

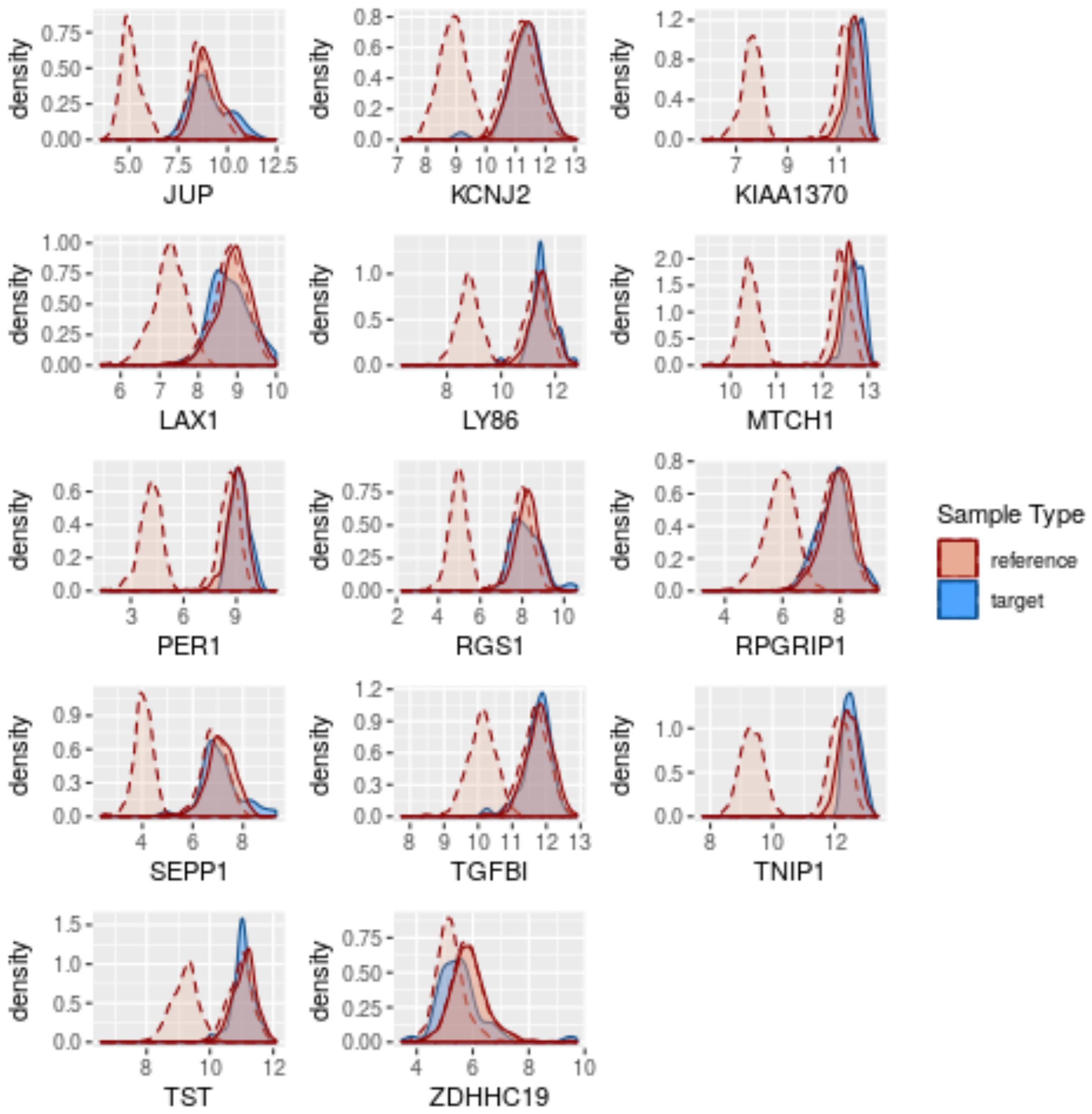
density
JUP
KCNJ2
KIAA1370
LAX1
LY86
MTCH1
PER1
RGS1
RPGRIP1
SEPP1
TGFBI
TNIP1
TST
ZDHHC19
Sample Type
reference
target

Supplementary Figure 3. Dimensionality reduction plots. (A,C) t-distributed stochastic neighbor embedding and (B,D) principal components analysis plots of IMX data. In (A,B) samples are colored by class, in (C,D) samples are colored by study. Both embeddings are based on the full set of 29 mRNAs, and show broad separability of the classes in high-dimensional space. There is residual study-to-study heterogeneity even after removal of technical heterogeneity by COCONUT.

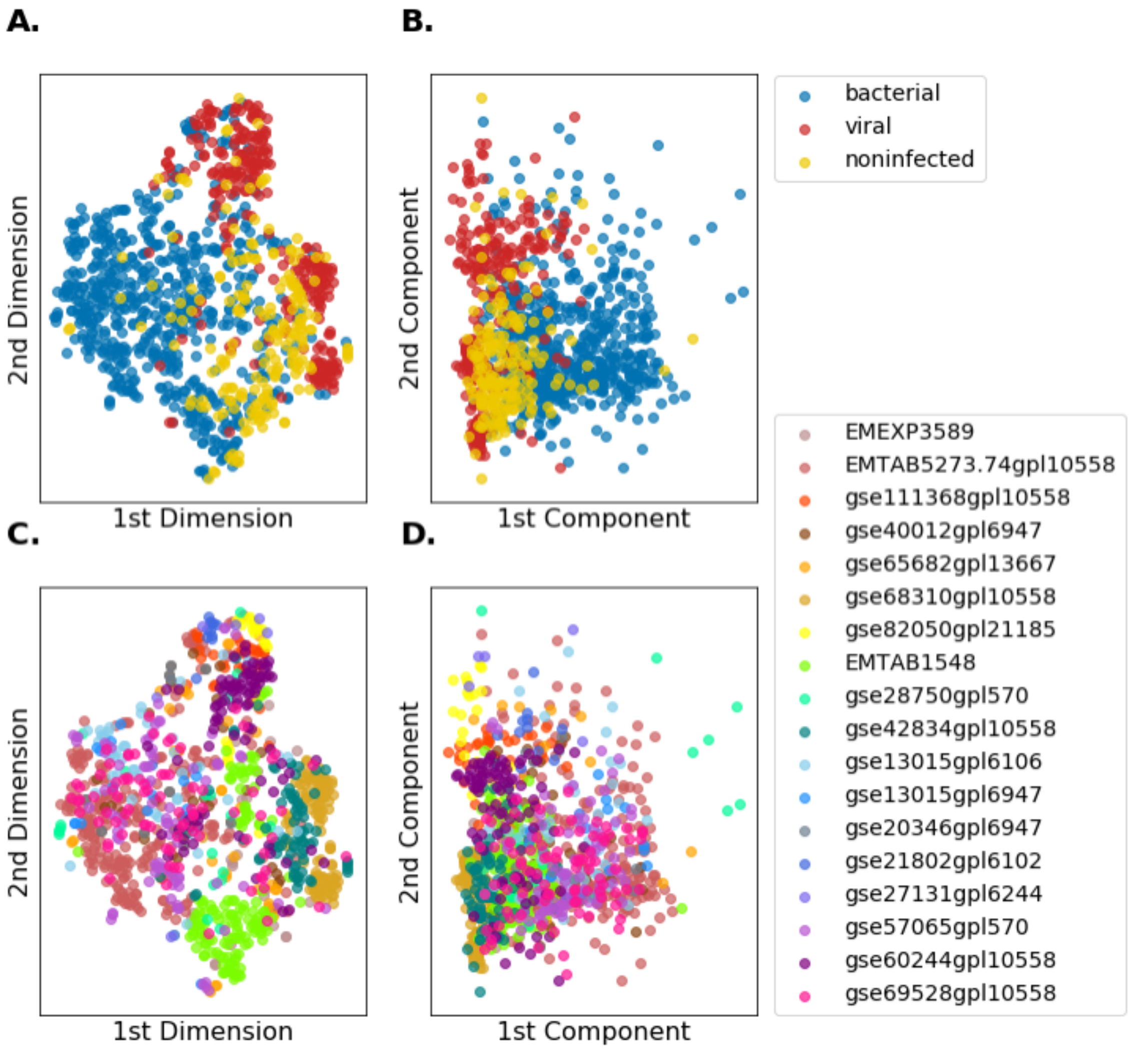

Supplementary Fig. 4. HiCV analysis of bias/overfitting using 29-mRNA expression vectors. A-C: logistic regression; D-F: SVM; G-I: XGBoost; J-L: MLP. Each row contains HiCV results for outer folds 1 (A, D, G, J), 2 (B, E, H, K) or 3 (C, F, I, L). The x-axis is the difference between outer fold APA and inner fold CV APA (a proxy measure for bias in generalization) for each combination of model and HiCV outer fold. The blue density plots correspond to this difference for the top 50 models ranked by LOSO CV on the inner fold. Orange density plots show this difference for the top 50 models ranked by 5-fold CV on the inner fold. The vertical dashed line indicates equality between inner fold and outer fold APA. The closer the density is to the dashed line, the smaller the difference between inner and outer fold performance for top classifiers identified by the given CV method. CV methods showing smaller differences between inner and outer fold performance (i.e. density closer to dashed line) might lead to selection of classifiers that generalize better to unseen data. Overlap between the density plots indicates that both CV methods (k-fold and LOSO) produce top classifiers with similar biases in performance between cross-validation on the inner fold and validation on the outer fold.

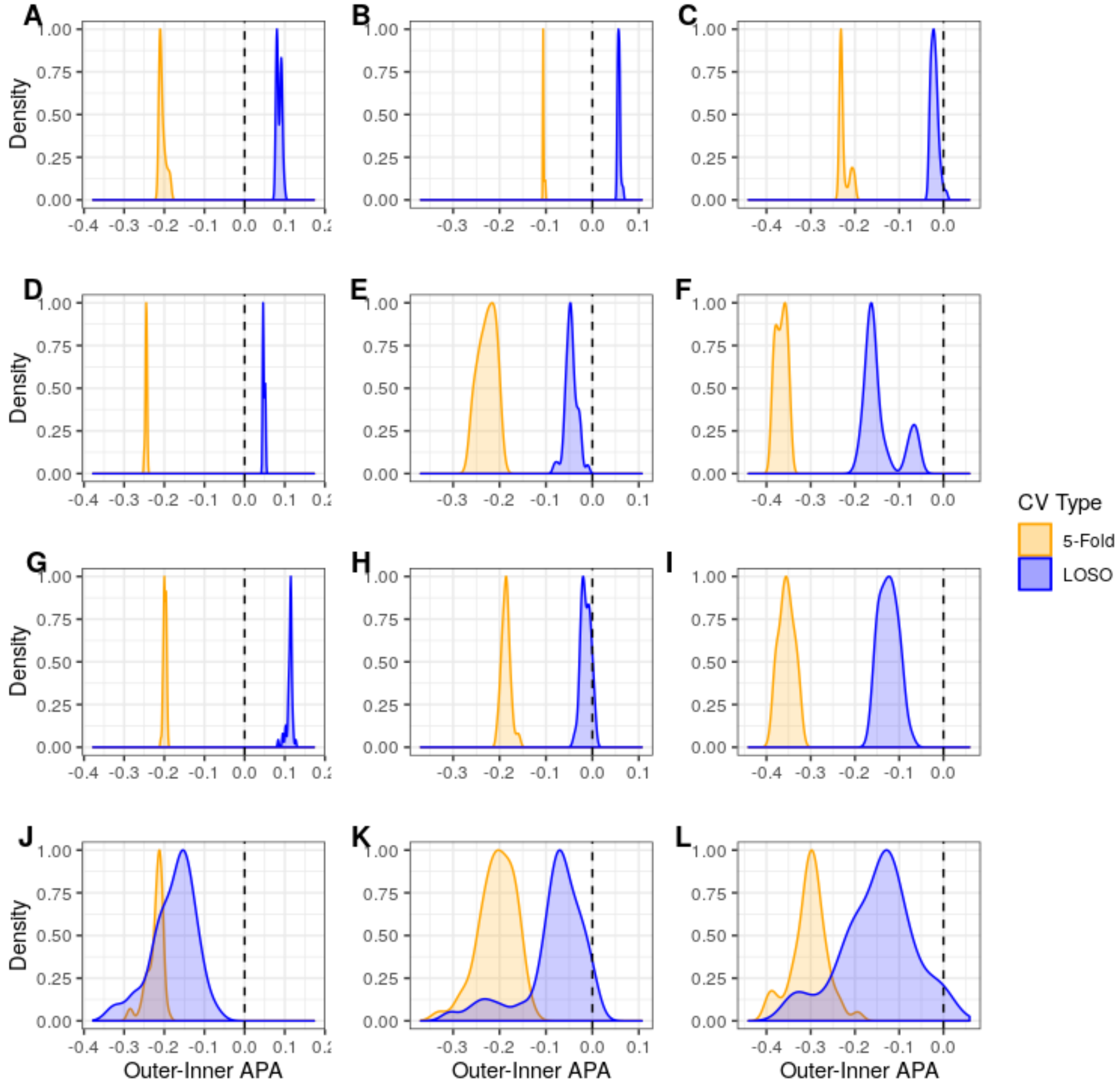

Supplementary Fig. 5. HiCV analysis of bias/overfitting of the top 10 classifiers as ranked by inner-fold performance using 6 GM scores. A-C: logistic regression; D-F: SVM; G-I: XGBoost; J-L: MLP. Each row contains HiCV results for outer folds 1 (A, D, G, J), 2 (B, E, H, K) or 3 (C, F, I, L). The x-axis is the difference between outer fold and inner fold APA; the y-axis corresponds to the outer fold APA. The vertical dashed line indicates equality between inner fold and outer fold APA. A classifier with high outer fold APA (y-axis) and nearly identical performance on the inner fold (x-axis near zero) is more favorable.

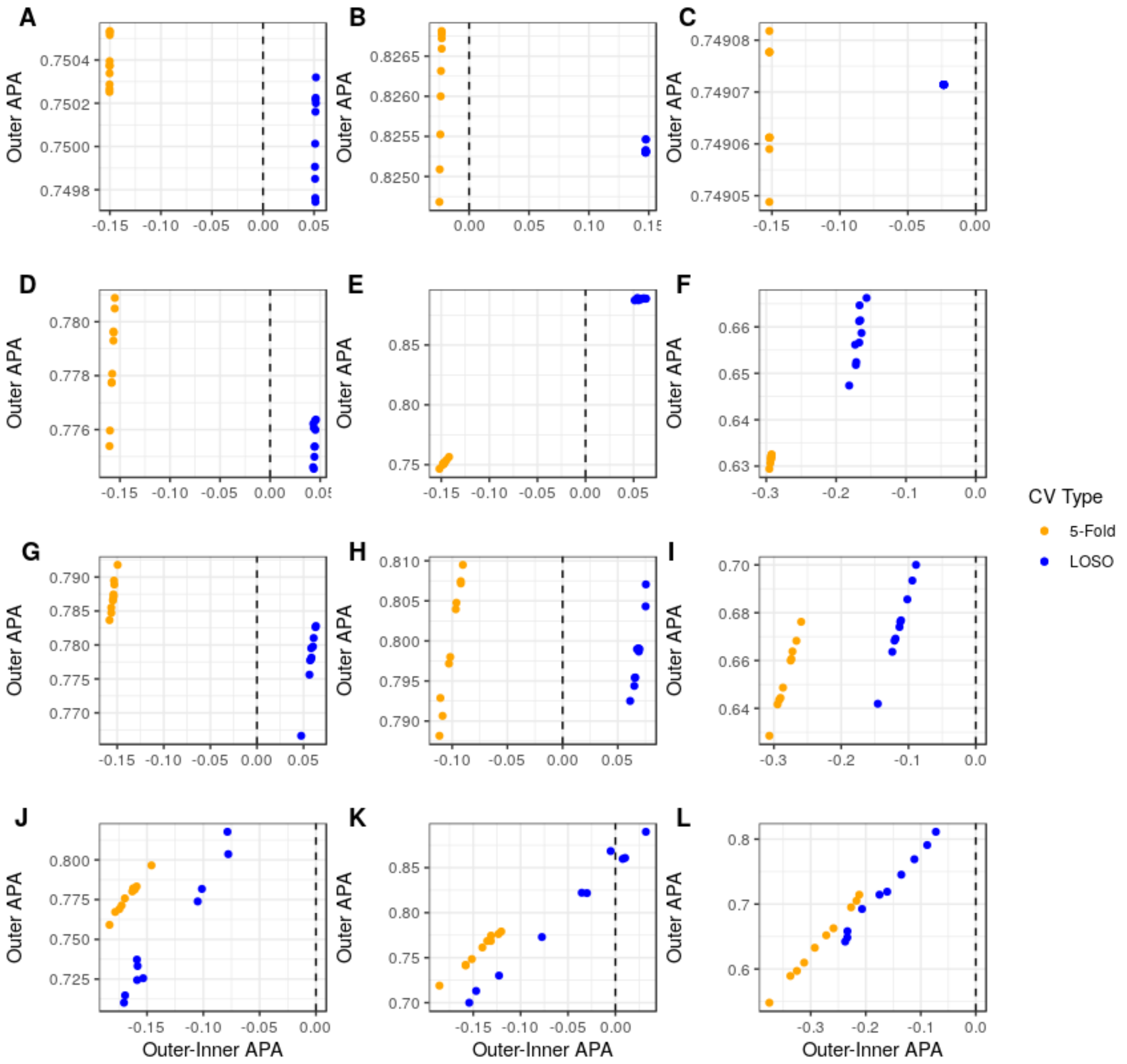

Supplementary Fig. 6. HiCV analysis of bias/overfitting of the top 10 classifiers as ranked by inner-fold performance using 29-mRNA expression vectors. A-C: logistic regression; D-F: SVM; G-I: XGBoost; J-L: MLP. Each row contains HiCV results for outer folds 1 (A, D, G, J), 2 (B, E, H, K) or 3 (C, F, I, L). The x-axis is the difference between outer fold and inner fold APA; the y-axis corresponds to the outer fold APA. The vertical dashed line indicates equality between inner fold and outer fold APA. A classifier with high outer fold APA (y-axis) and nearly identical performance on the inner fold (x-axis near zero) is more favorable.

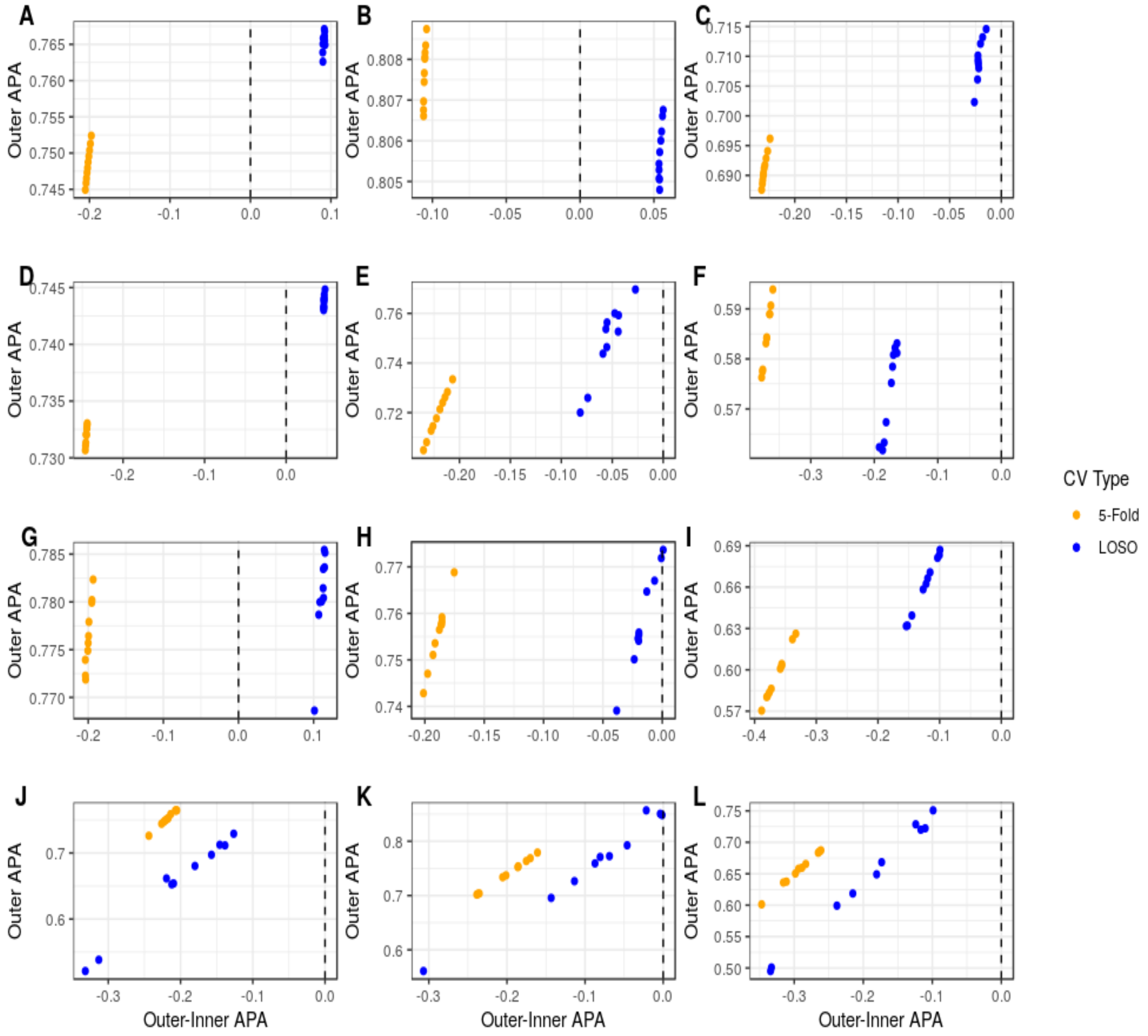

Supplementary Figure 7. Dimensionality reduction in Stanford ICU data. (A) t-distributed stochastic neighbor embedding and (B) principal components analysis plots of the Stanford ICU data. Samples are colored by class. Both embeddings are based on the full set of 29 mRNAs.

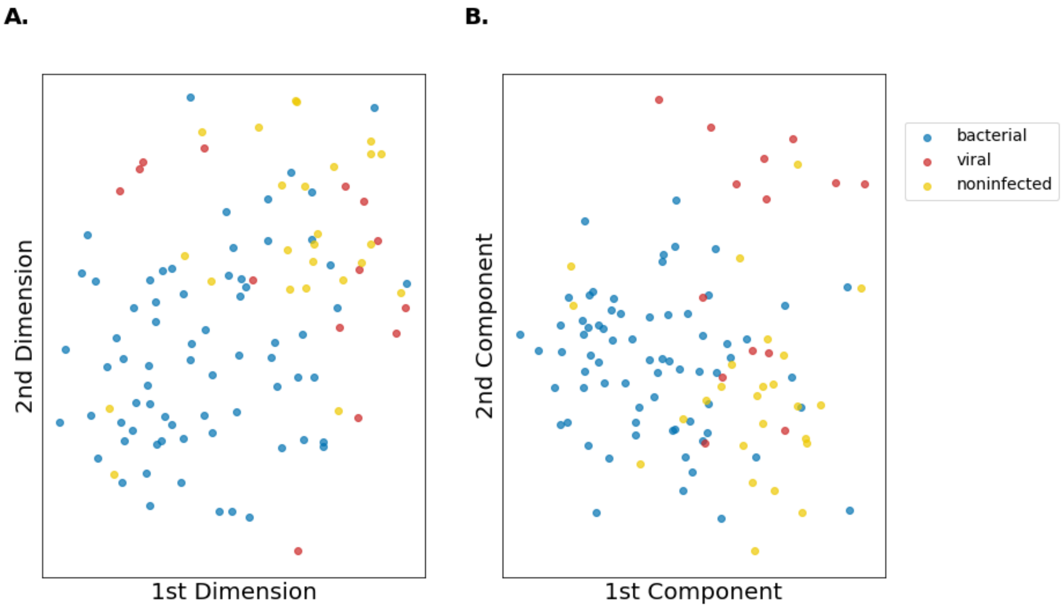

Supplementary Figure 8. Procalcitonin and C-reactive protein ROC plots in the Stanford ICU cohort. AUC = Area under the ROC curve.

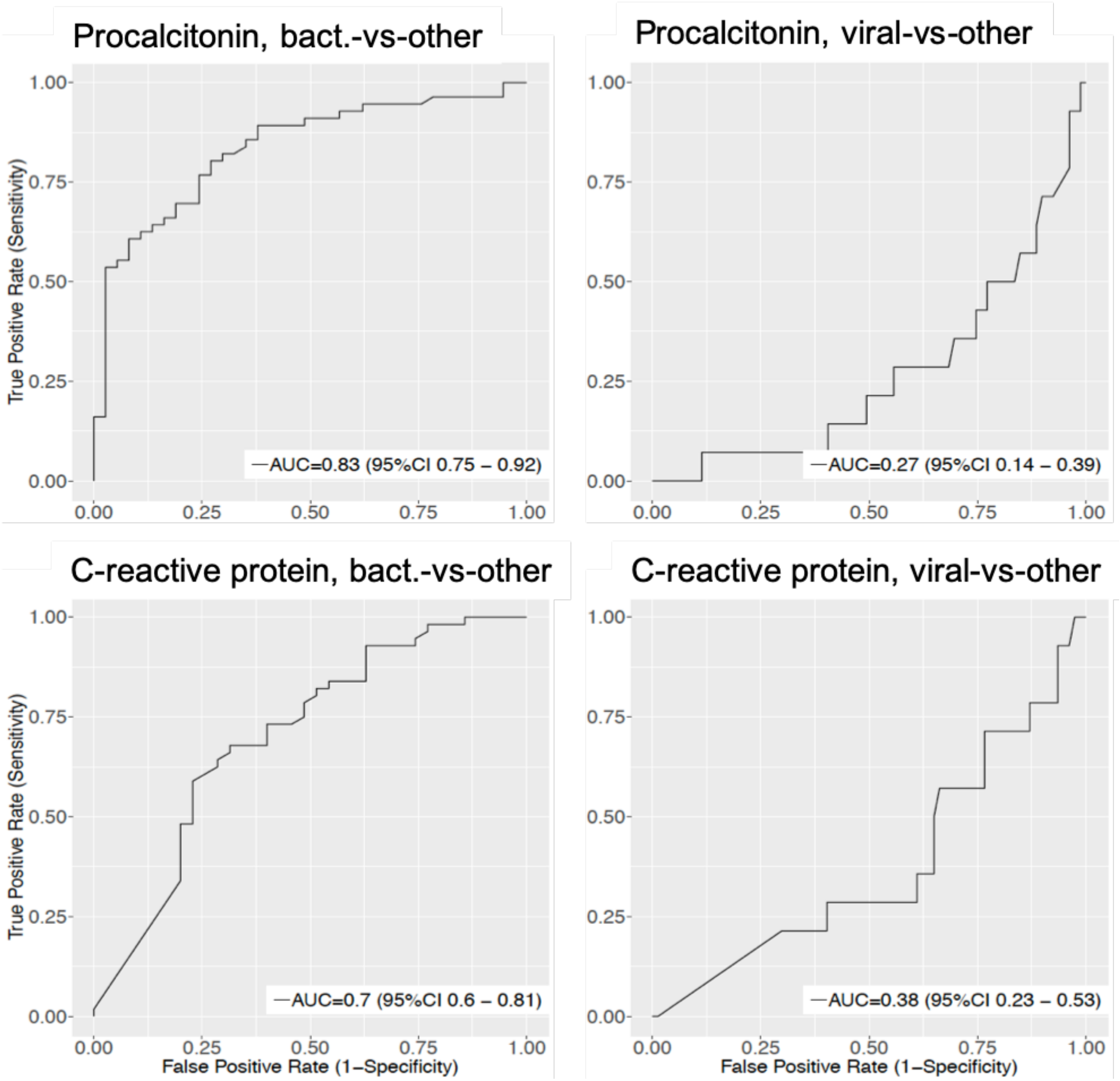

Supplement: Supplementary file 1 — Supplementary Information [file 41467_2020_14975_MOESM1_ESM.pdf]
