# Supplementary material for: A generalizable 29-mRNA neural-network classifier for acute bacterial and viral infections
Source: Nat Commun. 2020 Mar 4;11:1177. doi: 10.1038/s41467-020-14975-w (PMC7055276; doi:10.1038/s41467-020-14975-w)
Supplement: Supplementary file 2 — Description of Additional Supplementary Files [file 41467_2020_14975_MOESM2_ESM.pdf]

**Description of Additional Supplementary Files**

File Name: Supplementary Data 1

Description: Normalized NanoString data.
