# Supplementary material for: A generalizable 29-mRNA neural-network classifier for acute bacterial and viral infections
Source: Nat Commun. 2020 Mar 4;11:1177. doi: 10.1038/s41467-020-14975-w (PMC7055276; doi:10.1038/s41467-020-14975-w)
Supplement: Supplementary file 4 — Reporting Summary [file 41467_2020_14975_MOESM4_ESM.pdf]

nature research

Corresponding author(s): Timothy Sweeney

Last updated by author(s): Jan 21, 2020

Reporting Summary

Nature Research wishes to improve the reproducibility of the work that we publish. This form provides structure for consistency and transparency in reporting. For further information on Nature Research policies, see Authors & Referees and the Editorial Policy Checklist.

Statistics

For all statistical analyses, confirm that the following items are present in the figure legend, table legend, main text, or Methods section.

n/a	Confirmed	
☐	☒	The exact sample size (n) for each experimental group/condition, given as a discrete number and unit of measurement
☐	☒	A statement on whether measurements were taken from distinct samples or whether the same sample was measured repeatedly
☐	☒	The statistical test(s) used AND whether they are one- or two-sided<br>*Only common tests should be described solely by name; describe more complex techniques in the Methods section.*
☒	☐	A description of all covariates tested
☒	☐	A description of any assumptions or corrections, such as tests of normality and adjustment for multiple comparisons
☐	☒	A full description of the statistical parameters including central tendency (e.g. means) or other basic estimates (e.g. regression coefficient) AND variation (e.g. standard deviation) or associated estimates of uncertainty (e.g. confidence intervals)
☐	☒	For null hypothesis testing, the test statistic (e.g. F, t, r) with confidence intervals, effect sizes, degrees of freedom and P value noted<br>*Give P values as exact values whenever suitable.*
☒	☐	For Bayesian analysis, information on the choice of priors and Markov chain Monte Carlo settings
☒	☐	For hierarchical and complex designs, identification of the appropriate level for tests and full reporting of outcomes
☒	☐	Estimates of effect sizes (e.g. Cohen's d, Pearson's r), indicating how they were calculated

Our web collection on statistics for biologists contains articles on many of the points above.

Software and code

Policy information about availability of computer code

Data collection

The core software used in developing IMX-BVN-1 is described in the Supplemental Methods, including open source software tools. Further details are available on request. The proprietary portions of the code are not available.

There has been a precedent for allowing proprietary code and models to not be made publicly available. For instance, in Blauwkamp et al, Nature Microbiology 2019, proprietary code from Karius, Inc., was not made publicly available. Second, the particular code base used in this study is a relatively complex distributed software system not designed for sharing with outside users. Making it useable for other researchers such that they could exactly replicate our work would require substantial resources which are not available to the authors at this time.

That said, we have made every effort to describe the work in enough detail for a researcher to reproduce the methods contained herein so that our work can be built upon (e.g., extensive descriptions of model selection process, pseudocode of COCONUT matching), which is the main goal of public sharing.

Data analysis

See above

For manuscripts utilizing custom algorithms or software that are central to the research but not yet described in published literature, software must be made available to editors/reviewers. We strongly encourage code deposition in a community repository (e.g. GitHub). See the Nature Research guidelines for submitting code & software for further information.

Data

Policy information about availability of data

All manuscripts must include a data availability statement. This statement should provide the following information, where applicable:

- Accession codes, unique identifiers, or web links for publicly available datasets
- A list of figures that have associated raw data
- A description of any restrictions on data availability

Gene expression data are publicly available at their stated accession IDs (Table 1).

Field-specific reporting

Please select the one below that is the best fit for your research. If you are not sure, read the appropriate sections before making your selection.

☒ Life sciences ☐ Behavioural & social sciences ☐ Ecological, evolutionary & environmental sciences

For a reference copy of the document with all sections, see nature.com/documents/nr-reporting-summary-flat.pdf

Life sciences study design

All studies must disclose on these points even when the disclosure is negative.

Sample size	Training sample size was chosen by including all patient samples available after a methodical search of public data. Test data sample size was chosen as all samples available in a cohort study of appropriate enrollment criteria.
Data exclusions	Only samples which did not meet inclusion criteria are fully excluded from the manuscript. Samples of indeterminate class are excluded from the main analysis, per the Methods.
Replication	A fully independent test cohort (Stanford ICU cohort) is present and reported.
Randomization	N/A; this was a cohort analysis, not a randomized trial. Per standard in the field, diagnostic AUCs are presented for the index and reference diagnostics without covariate analysis.
Blinding	Samples were run blinded to adjudications, and adjudications were done blinded to novel diagnostic scores (double blind).

Reporting for specific materials, systems and methods

We require information from authors about some types of materials, experimental systems and methods used in many studies. Here, indicate whether each material, system or method listed is relevant to your study. If you are not sure if a list item applies to your research, read the appropriate section before selecting a response.

Materials & experimental systems

n/a	Involved in the study	
☒	☐	Antibodies
☒	☐	Eukaryotic cell lines
☒	☐	Palaeontology
☒	☐	Animals and other organisms
☐	☒	Human research participants
☐	☒	Clinical data

Methods

n/a	Involved in the study	
☒	☐	ChIP-seq
☒	☐	Flow cytometry
☒	☐	MRI-based neuroimaging

Human research participants

Policy information about studies involving human research participants

Population characteristics	We collected blood into PAXgene RNA tubes from 165 patients enrolled in the Stanford University Medical ICU Biobank from 2015-2018 (Stanford IRB #28205). Adult subjects enriched for acute respiratory distress syndrome risk factors (e.g. sepsis, aspiration, trauma) were recruited at admission to the Stanford ICU from either the hospital wards or the emergency department as part of an existing biobanking study. Patients eligible for inclusion were consecutive adults (>= 18 years) admitted to Stanford ICU with at least one ARDS risk factor (e.g. sepsis, pneumonia, trauma, aspiration). We excluded routine post-op patients, those admitted for a primary neurologic indication, and those with anemia (hemoglobin <8). Screening of consecutive new admissions via electronic medical records review of all ICU subjects was performed by a study coordinator and the study PI (AJR). Screening occurred on weekdays with a goal enrollment in <24h of admission to ICU, and included patients admitted to the ICU from the wards or the emergency room. Patients or their surrogates were approached for consent to participate in the Stanford ICU biobank, and the PAXgene tubes used for this study were collected between October 2015 to April 2017.

Recruitment	ICU patients meeting enrollment criteria were recruited at ICU admission.
Ethics oversight	Stanford University IRB (approval #28205)

Note that full information on the approval of the study protocol must also be provided in the manuscript.

Clinical data

Policy information about clinical studies

All manuscripts should comply with the ICMJE guidelines for publication of clinical research and a completed CONSORT checklist must be included with all submissions.

Clinical trial registration	N/a, cohort study using a prospective biobank, as described in methods.
Study protocol	present in the methods and supplemental methods
Data collection	Fully described in methods section; single-center biobanking study.
Outcomes	primary outcome was AUROC of IMX-BVN-1 in Stanford ICU cohort, as described in methods.
